# Differential projections from the cochlear nucleus to the inferior colliculus in the mouse

**DOI:** 10.3389/fncir.2023.1229746

**Published:** 2023-07-24

**Authors:** David K. Ryugo, Giedre Milinkeviciute

**Affiliations:** ^1^Garvan Institute of Medical Research, Darlinghurst, NSW, Australia; ^2^School of Biomedical Sciences, University of New South Wales, Kensington, NSW, Australia; ^3^Department of Otolaryngology, Head and Neck and Skull Base Surgery, St. Vincent’s Hospital, Darlinghurst, NSW, Australia

**Keywords:** anatomy, auditory, circuits, dextran amine, hearing, projections, tonotopy

## Abstract

The cochlear nucleus (CN) is often regarded as the gateway to the central auditory system because it initiates all ascending pathways. The CN consists of dorsal and ventral divisions (DCN and VCN, respectively), and whereas the DCN functions in the analysis of spectral cues, circuitry in VCN is part of the pathway focused on processing binaural information necessary for sound localization in horizontal plane. Both structures project to the inferior colliculus (IC), which serves as a hub for the auditory system because pathways ascending to the forebrain and descending from the cerebral cortex converge there to integrate auditory, motor, and other sensory information. DCN and VCN terminations in the IC are thought to overlap but given the differences in VCN and DCN architecture, neuronal properties, and functions in behavior, we aimed to investigate the pattern of CN connections in the IC in more detail. This study used electrophysiological recordings to establish the frequency sensitivity at the site of the anterograde dye injection for the VCN and DCN of the CBA/CaH mouse. We examined their contralateral projections that terminate in the IC. The VCN projections form a topographic sheet in the central nucleus (CNIC). The DCN projections form a tripartite set of laminar sheets; the lamina in the CNIC extends into the dorsal cortex (DC), whereas the sheets to the lateral cortex (LC) and ventrolateral cortex (VLC) are obliquely angled away. These fields in the IC are topographic with low frequencies situated dorsally and progressively higher frequencies lying more ventrally and/or laterally; the laminae nestle into the underlying higher frequency fields. The DCN projections are complementary to the somatosensory modules of layer II of the LC but both auditory and spinal trigeminal terminations converge in the VLC. While there remains much to be learned about these circuits, these new data on auditory circuits can be considered in the context of multimodal networks that facilitate auditory stream segregation, signal processing, and species survival.

## Introduction

The central auditory system is specialized to detect and analyze sounds distinguished by pitch (frequency content), cadence (e.g., prosody, melody, or timing), and location. The CN is the first central auditory nucleus to receive auditory information from the periphery. The DCN (or acoustic tubercle) and VCN represent the two main divisions of the CN on the basis of their internal cellular anatomy (Ramoìn y Cajal, 1909), physiological properties of the resident neurons (Pfeiffer, 1966; Evans and Nelson, 1973), multimodal inputs (Miller and Basbaum, 1975; Itoh et al., 1987; Weinberg and Rustioni, 1987; Wright and Ryugo, 1996; Shore and Zhou, 2006), and consequences of selective lesions (Masterton and Granger, 1988; Sutherland et al., 1998a,b). Both divisions are innervated by the auditory nerve, which bifurcates upon entering the CN to distribute information from the cochlea (Muniak et al., 2013). The DCN and its projections are involved in the analysis of spectral cues generated by reflected sound waves of the head and pinna. The nature of the reflections reveal important features of sound elevation, distance, and front-back distinctions (Sutherland et al., 1998a,b; Reiss and Young, 2005). The VCN is the gateway to all sound perception (Masterton and Granger, 1988; Masterton, 1997). One function of its synaptic connections involves sound localization in the azimuthal plane by mediating binaural differences in timing and intensity (Jeffress, 1948; Goldberg and Brown, 1969; Boudreau and Tsuchitani, 1970; Guinan et al., 1972; Yin and Chan, 1990; Park et al., 1996; Grothe, 2000; Konishi, 2000). Together, DCN and VCN initiates all ascending pathways in the central auditory system for subsequent analyses.

Sound cues, including frequency, intensity, and timing, are modified by the location of the sound source. Somatosensory, visual, and vestibular cues contribute to spatial processing by providing information about head, neck, and pinna position as well as by sound or listener’s movement (Middlebrooks, 2015; Yost and Pastore, 2019; Yost et al., 2020) which allow us to comprehend sound in space (Wright and Ryugo, 1996; Kanold and Young, 2001; Reiss and Young, 2005; Slee and Young, 2010; Wu and Shore, 2018; Yost et al., 2020). Air-borne sounds are also subject to the conditions of ambient air (wind, temperature, and humidity), environmental noise, and local ecosystems such as forest versus meadow versus burrow (Rossing, 1982; Haskell, 2022). Ascending and descending inputs from motor, somatosensory, proprioceptive, vestibular, and visual information converge in the IC (Aitkin et al., 1978, 1981; Robards, 1979; Ryugo et al., 1981, 2003; Björkeland and Boivie, 1984; Coleman and Clerici, 1987; Sparks and Hartwich-Young, 1989; Huffman and Henson, 1990; Bajo and King, 2013; Sekaran et al., 2021). The integration of multisensory information gives sound context and contributes to the spatial separation of concurrent sound streams. The circuits that convey these different modes of sensory information are key to understanding how mammals actively navigate through a complex and constantly changing acoustic environment while keeping sound source identity constant.

The unique properties attributed to VCN and DCN neurons (Pfeiffer, 1966; Evans and Nelson, 1973; Nelken and Young, 1994) appear characteristic for particular circuits (Grothe et al., 2010) and are hypothesized to underlie specific aspects of animal behavior (Sutherland et al., 1998a,b). A crucial feature of hypothesis building and modeling neuronal mechanisms of hearing is knowing how different sets of neurons are connected. The contralateral IC is one of the main targets of DCN and VCN projections, and their projections have been interpreted as overlapping with “similar properties” (Osen, 1972; Adams, 1979; Malmierca et al., 2005; Cant and Benson, 2006, 2008). Given the differences in the physiological responses of neurons in the VCN versus the DCN, however, we considered these circuit conclusions in conflict with concepts of structure and function. Our hypothesis was that the DCN and VCN should fundamentally differ in their pattern of connections to the IC.

The present study sought to determine how the pathways originating from the two CN divisions interact in the IC by mapping anterogradely labeled axons and terminals arising from the DCN and the VCN. We analyzed the organization of these terminations as well as their possible relationship with other sensory information processed in the IC to establish an anatomical foundation that will contribute to formulating mechanisms for the early stages of auditory processing.

## Materials and methods

### Animals

CBA/CaH mice (*n* = 20) and GAD67-EGFP (*n* = 4) of either sex and aged 2.5–5 months were used in this study. CBA/CaH mice have long been used in hearing research because they exhibit stable auditory brainstem response thresholds and minimal cochlear pathology over the first 2 years of life (Henry and Chole, 1980; Sergeyenko et al., 2013; Ohlemiller et al., 2016). GAD67–EGFP mice express enhanced green fluorescent protein (EGFP) under the glutamic acid decarboxylase 67 (GAD67) promoter in a C57Bl/6 background and are used to label neurons containing gamma-aminobutyric acid (GABA, Tamamaki et al., 2003). Animals were housed in the Biological Testing Facility of the Garvan Institute of Medical Research on a 12 h light/dark cycle and had *ad libitum* access to food and water. All procedures followed the animal care guidelines of the National Health and Medical Research Council and were approved by the Animal Ethics Committee of the Garvan Institute and St. Vincent’s Hospital, UNSW Australia.

### Auditory brainstem response

An auditory brainstem response (ABR) was recorded from each animal prior to surgery to verify normal hearing (Stamataki et al., 2006). Each mouse was anesthetized with an intraperitoneal injection of ketamine (100 mg/kg) and xylazine (10 mg/kg). When the animal was unresponsive to a toe pinch, it was placed in an electrically shielded, double-walled, sound-attenuated chamber padded with acoustic foam (Sonora Technology Co., Gotenba, Japan). ABR testing followed standard procedures of the lab as previously published (Connelly et al., 2017; Suthakar and Ryugo, 2017; Muniak et al., 2018). Only mice with click thresholds better than 30–40 dB SPL were included in these experiments.

### Surgery to implant headpost

Each animal was secured in a stereotaxic frame (Stoelting, Wood Dale, IL, USA) using ear bars and a bite bar. Body temperature was maintained at 37°C using an infrared heating pad. Anesthesia was maintained using isoflurane (1.5–2.0% in ∼600 cc/min O_2_). The skull landmarks bregma and lambda were surgically exposed, and a custom-made steel head post was cemented to the skull just rostral to bregma to stabilize the animal for later physiological recordings (Muniak et al., 2012) and a tungsten ground-pin was inserted into the skull nearby. A small craniotomy was made directly over the target structure (DCN or VCN), which was stereotaxically guided by a mouse brain atlas (Paxinos and Franklin, 2019). The craniotomy was covered with bone wax. The mouse given 1 cc of saline subcutaneously for rehydration and left to recover for 1 day prior to electrophysiological recordings and injection.

### Electrophysiological recordings and dye injection: VCN vs DCN

Recordings were performed in our above-mentioned sound-attenuated chamber. The mouse was lightly sedated with an intraperitoneal injection of acepromazine (0.07 mg/kg), placed in a plastic tube that restricted body movement, and secured by affixing the head post to a custom-built apparatus mounted within a stereotaxic frame (David Kopf Instruments, Tujunga, CA, USA). Bone wax was removed from the craniotomy just prior to recording. Quartz glass micropipette electrodes filled with 10% neuronal tracer dye [Alexa Fluor 488, Alexa Fluor 555, or biotinylated dextran amine (BDA) in a solution of 0.05M Tris Buffer and 0.15 KCl, pH 7.3 (Supplementary Table 1)], were used for multiunit recordings (inner tip diameter: 15–20 μm).

Stimulus delivery and neural recordings were controlled via custom software. Acoustic stimuli were generated digitally (DAP5016a, Microstar Laboratories, Bellevue, WA, USA), anti-aliased (Model 3202 Krohn-Hite, Brockton, MA, USA), amplified (Halo A23, Parasound, San Francisco, CA, USA), attenuated (PA5, TDT), and delivered by a calibrated free-field speaker (EMIT High Energy; Infinity, La Crescent, MN, USA) placed 10 cm from the mouse and 25° off the midline. Neural signals were amplified and filtered (2400A; Dagan, Minneapolis, MN, USA), passed through a spike signal enhancer (40-46-1; FHC, Bowdoinham, ME), and digitized for analysis (DAP5016a; Microstar Laboratories). 200 ms broadband or sinusoidal tone search stimuli (4/sec) were delivered as the recording electrode was advanced into the brain using a motorized hydraulic micromanipulator (Model 2650; David Kopf Instruments).

Entry into the VCN or DCN was marked by the unambiguous presence of sound-evoked spike discharges. A frequency response area was obtained using tone bursts at 5dB variable intensities swept through the mouse’s audible range (∼4–100 kHz). A site was chosen that gave a strong evoked response as determined audiovisually by manually adjusting the tone burst frequency and attenuation. Characteristic frequency (CF) for the site was confirmed with an automated tuning curve protocol (MATLAB, MathWorks, Natick, MA, USA) that measured responses to a 4-octave (oct.) sweep centered on the test frequency at 20 dB above threshold, sampling every 1/25-oct (Muniak et al., 2013). With these frequency data, the neuronal tracer was deposited iontophoretically using a high voltage, constant current source (CS 3; Midgard/Stoelting) set at 5 μA and 50% duty cycle for 5–10 min into the site giving the best frequency-evoked response. On several occasions, two injections were attempted at 2 different frequency sites in the same nucleus. After a rest period of 5 min the electrode was withdrawn, the craniotomy covered with bone wax, and the mouse returned to its cage. A survival period of 10–18 days ensured adequate filling of neuronal tracers.

### Tissue processing

Animals were deeply anesthetized via a lethal dose of sodium pentobarbitone (100 mg/kg, intraperitoneally), and perfused transcardially with 5 ml 1% sodium nitrite prewash in 0.1M phosphate buffered saline followed immediately by 60 ml 4% paraformaldehyde/0.1% glutaraldehyde in 0.1M phosphate buffer. Heads were postfixed overnight after which the brain was dissected from the skull, embedded in gelatin-albumin, and cut in the transverse plane at 50–60 μm using a vibrating microtome (VT1200S; Leica Systems, Nussloch, Germany). All reagents and rinses were made up in 0.12M Tris-buffered saline.

Visualization of Alexa Fluor 488 or 555 did not require any further tissue processing. Coronal sections were mounted and coverslipped using Vectashield (H-1400; Vector Labs) and viewed with a fluorescent microscope. To visualize BDA labeling for brightfield and electron microscopy, sections were incubated for 10 min in 1% H2O2, rinsed 3×, permeabilized for 1 h in 0.5% Photo-Flo (Kodak, Rochester, NY, USA) or 0.5% Triton X-100 (brightfield only), and then incubated in ABC (Vectastain Elite ABC Kit, PK-6100; Vector Labs) with 0.5% Photo-Flo/Triton for 1 h. The tissue was rinsed 3× again and BDA labeling was developed using nickel-intensified diaminobenzidine (DAB; Sigma-Aldrich). Sections were mounted, dehydrated, and coverslipped with Permount (Fisher Scientific, Pittsburgh, PA, USA).

### Tissue analysis

Photomicrographs were collected from a Zeiss Axioplan microscope equipped for brightfield and fluorescent imaging with Plan Neofluar Objectives (20×/0.50, 40×/0.75, and 100×/1.25 oil), and standard light microscopic methods were applied to both brightfield and fluorescent images (Muniak et al., 2013; Muniak and Ryugo, 2014; Williams et al., 2022). Labeled fibers and terminals were manually traced from digitized photomontages with a graphics tablet (Cintiq 22HD; Wacom, Portland, OR, USA) to optimize details.

### Cochlear nucleus and injection site reconstructions

Serial coronal sections of the entire left CN were photographed using a 10× objective and photomontages were produced by manually aligning digitized sections using *Adobe Photoshop 2021*. The granule cell domain (GCD) was used to separate the DCN from the VCN (Mugnaini et al., 1980a; Muniak et al., 2013). The DCN and VCN borders and the injection site were outlined in consecutive photographs using a graphic tablet and *Photoshop 2021* for each mouse. The area of the injection site was determined by outlining the densest accumulation of DAB reaction product or border of fluorescence at medium illumination. The outlines were mapped as an image-stack to identify the approximate geometric center of the injection site, which was then photographed. A single injection site was recovered in the VCN of 6 mice and in the DCN of 13 other mice; 2 injections were recovered in one DCN of a single mouse. Data from these mice form the basis of this report.

### Axon tracing

**FIGURE 1:**
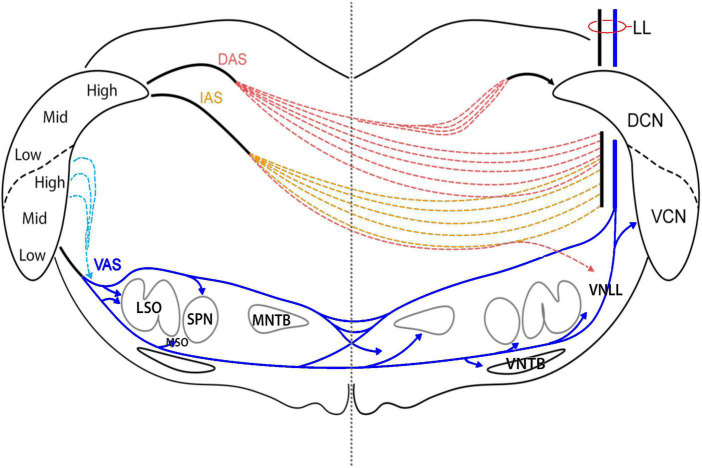
Diagram of the acoustic striae as they leave the CN and traverse the brainstem. Each of the striae leaves the CN as a discrete bundle but breaks down after 100 μm as individual fibers begin to travel independently. DAS fibers separate as they pass medial to the superior vestibular nucleus. After crossing the midline, a branch of fibers reconvenes in the contralateral DAS and runs medial to the DCN to enter the CN via the subpeduncular corner. Fibers from the anterior DCN and posterior VCN exit by taking a dorsomedial route via the IAS. These fibers cut through the vestibular nerve root and the spinal tract of the trigeminal as they travel ventrally toward the midline. Once past the midline, they continue laterally to enter the LL. A small group of fibers continue ventrally to enter the VNLL. Most of the fibers of the VCN exit by way of the ventral acoustic stria, also known as the trapezoid body. Some fibers from the posterior and dorsal regions of the VCN exit straight through the spinal tract of the trigeminal before looping downward to join the TB. Just lateral to the SOC, most TB fibers go over or under the LSO; some enter the LSO. Fibers leave the TB to innervate the ipsilateral SPN and MSO, as well as the contralateral MNTB, MSO, and VNTB before entering the LL.

Digital imaging facilitated the mapping of axons as they ascended the lateral lemniscus (LL) to enter the contralateral IC. Using a digital drawing tablet, axons leaving the injection site were traced through serial sections of the dorsal, intermediate, and ventral acoustic striae (DAS, IAS, and VAS, respectively) of the CN and across the brainstem to the contralateral LL (Figure 1). The principle is similar to *camera lucida* style drawings using a microscope drawing tube attachment. These axons did not stay as a tight bundle but scattered with an overall trajectory that is consistent with textbook illustrations (Carpenter, 1978).

### Axon ramifications and terminal fields

The boundary of the IC and the approximate subdivisions are shown in Supplementary Figure 1 (criteria of Lesicko et al., 2016; Milinkeviciute et al., 2017; Weakley et al., 2022). Photographic tile sets that encompassed the brainstem, LL, and IC were collected using 10× and/or 20× objectives and were manually montaged (Supplementary Figure 2). The 10× images were resampled at 800 dpi and then digitally magnified so that individually labeled fibers could be manually drawn.

Drawings were also conducted on images collected using 10×, 20×, and 40× objectives through the terminal fields of CN projections (Supplementary Figure 3). The digitized drawings collected using different magnification objections were compared and it was determined that images collected at 400 dpi and with a 10× objective suffered no significant loss of accuracy and greatly accelerated progress.

Manual drawing of labeled fibers and terminals was also performed on fluorescent images to illustrate patterns of terminations without interference from background fluorescence (Supplementary Figure 4). Using *Adobe Photoshop 2021*, we traced individual fibers on a different image layer on the graphics tablet at high magnification in high resolution images and then transferred just the drawing and IC outline to a new image (Supplementary Figure 4, panel 2C). This drawing method was key for showing global patterns of projections with low magnification images.

### Terminal analysis

Photographic z-stacks were collected through the middle of the terminal fields in 2 adjacent sections for analysis of the terminal endings. A rectangular box was placed over the hypothetical isofrequency laminar line in the CNIC and parallel to the laminar axis (see Supplementary Figure 5A, arrows). The image was digitally magnified such that 10 μm measured 10 mm on the digital drawing tablet. All distinct swellings (overlapping swellings were not included) were drawn using a digital pen having a 2 pixel tip (on a 400 dpi image). As previously noted in cats and rats (Malmierca et al., 2005), large and small terminals dominated the terminal field. Drawing and measuring terminal silhouettes were performed from digital images and analyzed. The size distinction could be qualitatively resolved during drawing, given different colors, and placed on separate layers. Drawn terminals were placed on a lined grid, with straight lines separated by 10 μm. For each terminal, the size (μm^2^) and perpendicular distance to the central *laminar* line could be assessed (Supplementary Figure 5B).

Terminals were also analyzed with respect to their location in the CNIC, DC, LC, or VLC. Boxes 170 μm x 200 μm were placed over the CNIC, whereas boxes 100 μm x 100 μm were placed over DC, LC, and VL to obtain an estimate of terminal patterning. All subdivisions of the IC exhibited a skewed distribution with respect to terminal silhouette area: there were many more small terminal endings than large ones. Terminal counts and density were not calculated because they are subject to uncontrollable factors including size and placement of the dye injection, dye transport efficacy, histological processing, and individual mouse differences.

## Results

The goal of this report is to describe new observations on circuits between the CN and the IC in the CBA/CaH mouse. We used iontophoretic injections of anterograde dyes at electrophysiologically defined frequency regions in either the DCN (*n* = 14) or the VCN (*n* = 6). Only animals with histologically verified injection sites are included in this report. While the central core of the injection sites was clearly located in either the DCN or the VCN, a halo indicative of some spread of the dye could sometimes be seen in the fiber pathway adjacent to the medial border of the CN: the inferior cerebellar peduncle (ICP), the descending sensory tract of the trigeminal (DST5), or the vestibular nerve root (VN). Since none of these structures have known connections with the IC, such possible contamination was not an issue. Fibers observed in the ipsilateral cerebellum and superior vestibular nucleus were likely caused by this spread.

### The acoustic striae

Ascending projections of the CN exit through well-known acoustic striae (Adams and Warr, 1976; Cant and Benson, 2003) but within a few hundred micrometers, begin to disperse (Figure 1). Axons leaving the injection site in the VCN traveled ventrally and medially to enter the ventral acoustic stria (VAS), also known as the trapezoid body (TB), along the medial border of the nucleus. The main branch of fibers continued in the TB toward the LSO. Since some VCN neurons project to the DCN and also have collaterals traveling through the TB, DCN injections will result in axonal labeling in the TB. The TB forks into dorsal and ventral streams at the lateral edge of the LSO, whereas some fibers continue straight to enter the nucleus. Their termination in the LSO defined a laminar sheet that was tonotopic and topographically related to the injection site (Doucet and Ryugo, 2003; Gómez-Álvarez and Saldaña, 2016; Williams et al., 2022). Fibers from the dorsal stream entered the ipsilateral superior paraolivary nucleus (SPN) and formed vertical sheets. Fibers from the ventral stream terminated around the ipsilateral medial superior olive (MSO) between the LSO and SPN. Past the midline, fibers from each stream entered and terminated in the contralateral MNTB. The terminations were tonotopic with high frequency fibers distributed medially and lower frequencies located more laterally. A dorsal stream passed over the top of the MNTB, gave off a few collaterals into the periolivary region, and entered the LL. The ventral stream continued as the TB and sent collateral branches and terminals into the contralateral MSO, the contralateral VNTB, and the contralateral VNLL where some fibers gave rise to calycine terminations. The remaining fibers entered the LL.

Fibers from the posterodorsal VCN and DCN fibers from the low frequency region form the intermediate acoustic stria (IAS). This bundle had a shallow arc that passed upward and medially to penetrate the inferior cerebellar peduncle (ICP), and then bent downward over the internal part of the solitary nucleus. These fibers crossed the midline in the central region of the brainstem and scattered while still maintaining a lateral and now upward arc. Some IAS fibers passed over and around the SOC where they dropped collaterals off into the resident SOC nuclei and the VNLL; the remaining fibers curved upward to enter the LL. Some of the fibers passed through the LL to enter the contralateral CN.

Projecting fibers from pyramidal and giant cells of the DCN entered the DAS from the fiber layer that formed the deep border of the nucleus. The DAS travels dorsally, medially, and anteriorly, looping over the ICP and curving through the medial vestibular nucleus (MVN). Fibers start to scatter while still maintaining the general trajectory to cross the midline through the central region of the brainstem, passing through the facial nerve root after the genu and the medial longitudinal fasciculus. Once past the midline, some fibers arc backward and upward into the contralateral DAS to enter the CN, whereas other continue their downward and anterior trajectory over the SOC to enter the LL.

Ventrotubercular axons of VCN planar stellate neurons were retrogradely labeled following dye injections in the DCN (Doucet and Ryugo, 1997; Doucet et al., 1999). These axons descended along the medial border of the CN to a branch point near the ventromedial base of the CN. One branch traveled laterally to the parent planar stellate neuron, whereas the other branch continued ventromedially in the VAS to join the trapezoid body (Doucet and Ryugo, 2003; Darrow et al., 2012). It is important to remember that these axons arise from the VCN, so their terminations are not part of the DCN system.

### Lateral lemniscus

**FIGURE 2:**
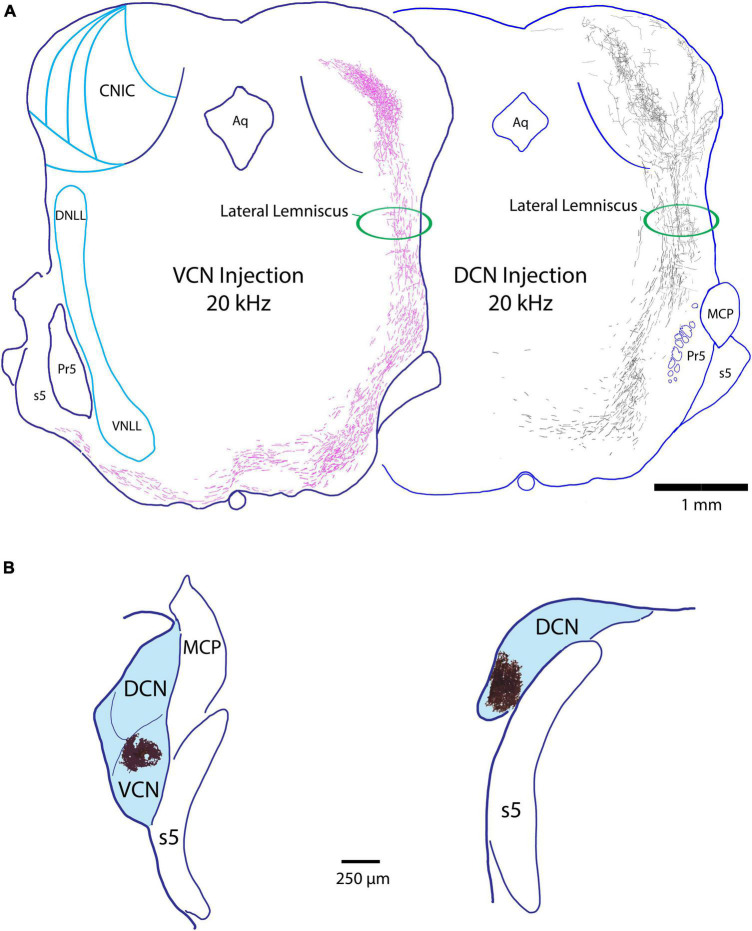
**(A)** Z-stack drawings through serial sections showing the course of fibers through the lateral lemniscus from a VCN projection (pink fibers, 20 kHz) and a DCN projection (black fibers, 20 kHz) as they ascend to the IC. Note that the fibers occupy separate corridors of the lemniscus, with VCN fibers situated laterally and DCN fibers medially. This spatial separation occurs irrespective of the frequency sensitivity of the fibers. We were unable to determine if the fibers have a tonotopic trajectory within the separate corridors of the LL. The horizontal fibers in the LL are collaterals of the ascending projections that terminate in the NLL. **(B)** Injection sites of BDA (magic-wand selection from tissue shown in dark brown) from the mouse cases shown in panel **(A)**, placed on schematic outlines of the respective VCN (left) and DCN (right). Aq, aqueduct of Sylvius; MCP, middle cerebellar peduncle; Pr5, principal nucleus of the trigeminal; s5, sensory root of the trigeminal.

As the LL ascends from the SOC to the IC, it forms a gentle curve that bends 1 mm anteriorly as it moves dorsolaterally and then back to enter the IC along its ventrolateral edge. Several features of the LL are distinguished: the ascending fibers from the contralateral DCN and VCN maintain relatively separate positions: the VCN fibers ascend in a lateral position, whereas the DCN fibers maintain a medial position (Figure 2). These fibers do not appear to establish a tonotopic position as they ascended in their respective corridors. The ascending fibers from both the VCN and DCN emit horizontal collaterals that travel to the different nuclei of the lateral lemniscus. There is a very minor ipsilateral projection from the CN to the IC: we observed 0–3 fibers from the ipsilateral VCN entering the IC per animal and none were observed from the ipsilateral DCN.

### Terminal fields in the IC

#### VCN projections

**FIGURE 3:**
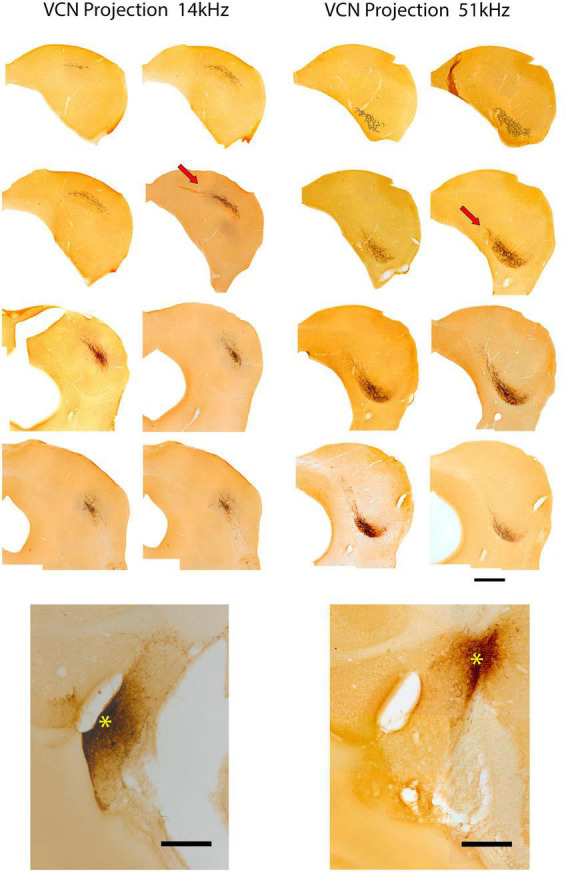
Photomicrographs through 2 series of IC sections after an injection of BDA into the VCN of separate mice and chromogenic processing with DAB. These two cases are representative of VCN injections with respect to pattern of terminations in the IC. The bottom panel of the left column shows the injection site (*) with a 14 kHz CF. Above is the corresponding series of alternate sections through the IC illustrating the location of the terminal field, characterized by darkly labeled fibers and terminals in the CNIC (red arrow illustrates one such field). The bottom right panel shows the injection site (*) in the VCN of a second mouse with a 51 kHz CF. Above is the corresponding series of alternate sections showing the terminal field in the contralateral CNIC. The location of the terminal field in both cases is relatively constant within the three-dimensions of the IC: the 14 kHz region defines a sheet of label more dorsally situated compared to that of the 51 kHz sheet. Frequency-specific projections from the VCN to the CNIC are topographic and define relatively narrow and restricted terminal fields. Scale bars: 500 μm.

VCN fibers of the LL continue their dorsal trajectory once inside the IC along the border of the CNIC and LCIC. At their appropriate tonotopic location [also illustrated by Williams et al. (2022)], the fibers make an oblique bend medially and dorsally into the CNIC, then ramify and arborize to form a flattened terminal field roughly 200 μm thick (Figure 3, arrows). Across serial sections, the layered arborization begins small posteriorly, grows in length as it moves more anteriorly, and then shortens again at the rostral end of the CNIC, forming a 3-dimensional disk. Medial extensions of the fibers can trickle into the lateral aspect of the DC. Two representative examples of VCN projections to the IC are presented in Figure 3. Alternate sections are shown going across from upper left (anterior) to lower right (posterior). This pattern is typical of all six of our cases with dye injections in the VCN.

#### DCN projections

**FIGURE 4:**
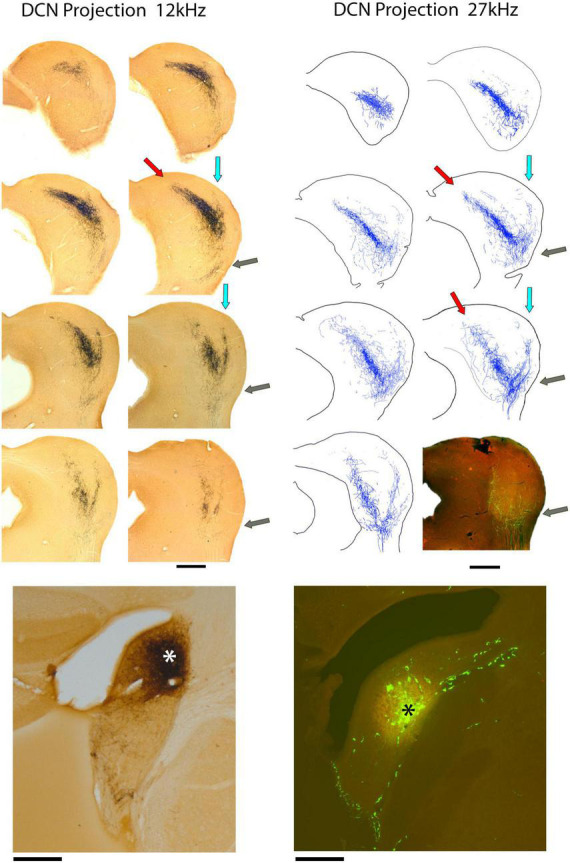
Photomicrographs **(left)** and drawings **(right)** through 2 series of IC sections after an injection of BDA into the DCN of separate mice and chromogenic processing with DAB. The bottom of the left column presents a photomicrograph of the 12 kHz injection site in the anterior DCN (*); the bottom of the right column shows the injection site at 27 kHz in the mid-DCN (*). Both injections are representative of all DCN projections, varying only in their topographic location. Each projection defines a three-pronged terminal field: the classic V-shaped, isofrequency projection with the primary (medial) limb distributed within the CNIC and DC (red arrows) and a dorsolateral limb traveling within layer III of the LC (blue arrows). The lateral limb of the “W” is distributed within the ventrolateral region of the lateral cortex, sometimes called the ventrolateral nucleus (gray arrows). This lateral limb projection is consistent but not heavy and is difficult to see at low magnification. Scale bars: 500 μm.

DCN fibers enter the IC from the medial aspect of the LL and some fraction of them promptly branch and project dorsolaterally into the VLC of the LC (Figure 4, gray arrows). This region is part of what has been called the external nucleus (Ramoìn y Cajal, 1909) or the ventrolateral nucleus (Morest and Oliver, 1984). What was called the lateral part of the CNIC in the cat (Morest and Oliver, 1984) is called the LC in the mouse because entering LL fibers run along the border between the CNIC and LC (Supplementary Figure 1; Ryugo et al., 1981; Lesicko et al., 2016; Milinkeviciute et al., 2017; Weakley et al., 2022).

Not all DCN fibers branch here because the number of entering fibers in the VLC is much smaller than those continuing to the CNIC and LC. The fibers entering VLC exhibit *en passant* swellings and branches that terminate as small sprays with terminal endings. Their density, however, is distinctly lower than that of the main projections. Fibers across the frequency range contribute to this small region of the IC in what appears to be tonotopic.

**FIGURE 5:**
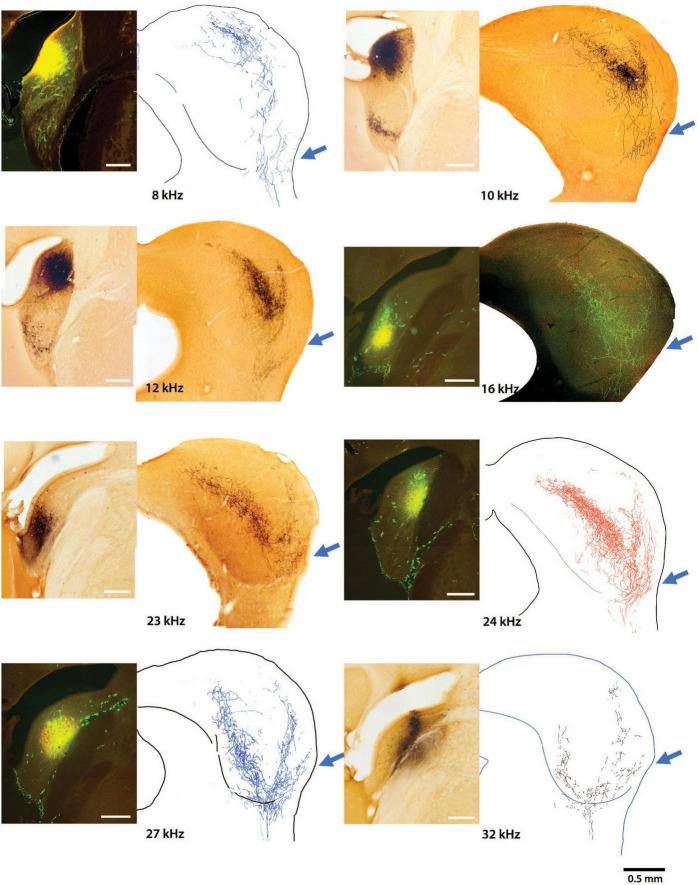
Photomicrographs of 8 injection sites in the DCN are paired with their corresponding mid-IC drawing or micrograph showing the tonotopic projections to the IC. The frequency response at each injection is also provided. The three-pronged terminal fields are evident in all the projections with attention given to the ventrolateral wing (arrow), which has not been previously described. Low frequency projections are located dorsally with higher frequency projections located progressively more ventrally. The terminal field of any particular frequency projection in the CNIC will exhibit overlap at the **top** and **bottom** surfaces of adjacent isofrequency contours. The medial extent of the CNIC projection extends a variable but distinct distance into the DC, maintaining tonotopic order. Terminal endings were distributed in the DCIC, CNIC, LCIC, and VLIC.

The main group of fibers rises along the medial border of the LC. Consistent with the tonotopic organization, the trunk forks to form two prominent branches. One bends dorsally and medially into the CNIC (Figure 4, red arrows) and clearly conforms to the tonotopic organization of the nucleus (Stiebler and Ehret, 1985; Williams et al., 2022). In contrast, the other branch maintains a lateral position and bends with layer III of the LC, following the contours of the IC surface (Figure 4, light blue arrows). The wings of this main projection form a prominent “V.” Injection sites appeared roughly equivalent for the VCN and DCN and yet, the projections from the DCN into the IC are consistently more prominent. The presence of the small projection into the VLC was observed in every DCN mouse, regardless of the injection size or CF taken. The pattern of topographic and tonotopic projections from the DCN to the IC is illustrated for 8 mice, accompanied by their injection sites (Figure 5).

**FIGURE 6:**
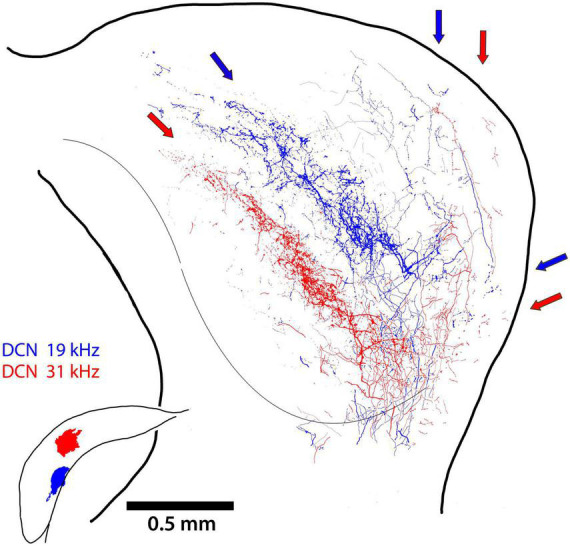
Two terminal fields of DCN axons from 2 different frequency locations (19 and 31 kHz) are illustrated for the same mouse. The main projection into the CNIC is clearly topographic, with some fibers continuing into the DC (red and blue arrows). There is also the dorsal branch that run up layer III of the LC while avoiding layer II and the somatosensory modules. The small lateral projection can also be seen the ventrolateral region of the LC.

In one mouse, we had separate injections in the DCN at two different frequency sites. The projecting terminals from the lower frequency location (19 kHz; Figure 6, blue arrows) are nested within the terminal field of the higher frequency projection (31 kHz; Figure 6 red arrows). The tonotopic relationship in a single mouse controls for inter-animal variability and differential distortion and shrinkage from tissue processing. This result prompted an examination of terminal field nesting across different animals.

**FIGURE 7:**
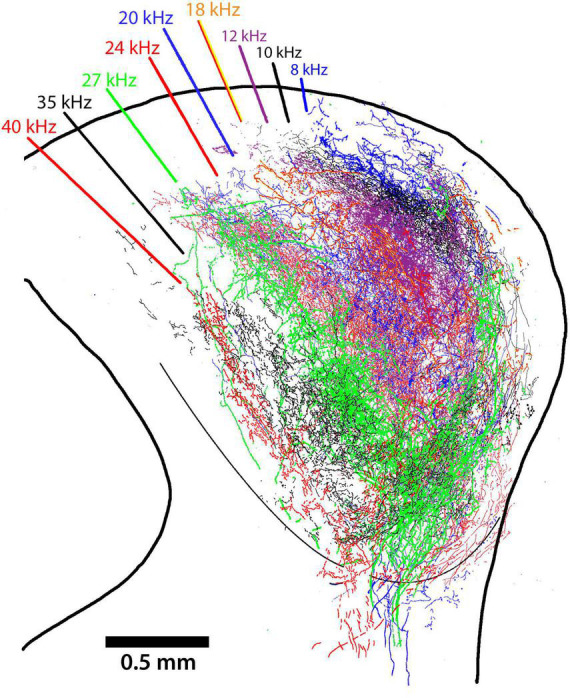
Tracings from photomontages through the middle of the CNIC illustrate the nested and tonotopic organization of DCN projections. The sections were adjusted only to match scale bars and to superimpose the midlines; the alignment by frequency is striking, especially in view of combining data across animals. The different frequencies are labeled and color-coded. The middle of each projection lamina has the heaviest concentration of terminals but there is overlapping scatter at the high and low frequency borders.

The middle section of the IC for nine mice with DCN injections was scaled and aligned to one another by referencing the IC surface to the ventricular aqueduct and stacked so that terminal fields representing different frequency could be assessed. In spite of individual differences in the size and shape of the ICs and the variations in the size and placement of dye injections, the stacked nesting of frequency projection fields in the CNIC is a prominent feature (Figure 7).

### Tonotopy

**FIGURE 8:**
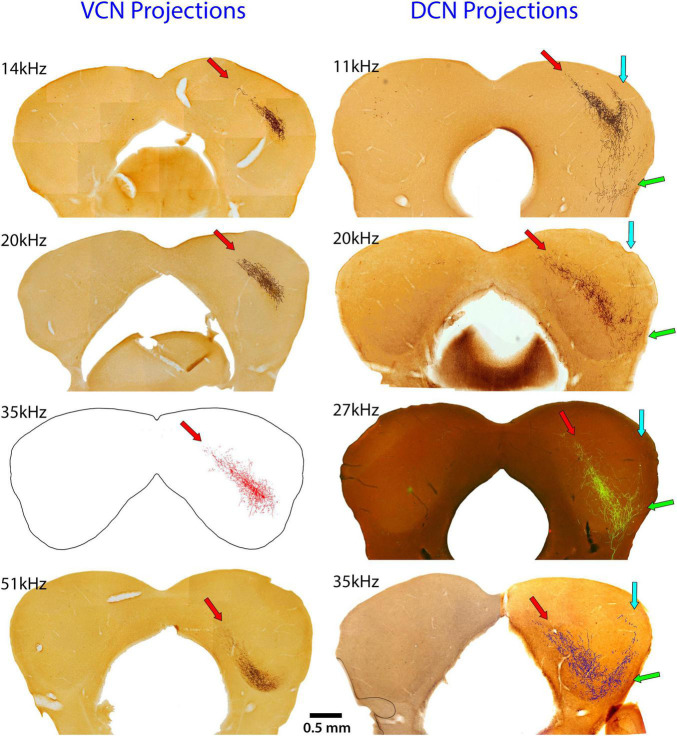
These images compare the termination patterns of VCN and DCN projections. The **left** column shows the terminal field in the contralateral CNIC (red arrow) of the VCN and the **right** column shows the contralateral terminal fields of the DCN. Note the shared and distinctly different projection terminations. Red arrows show the CNIC; blue arrows show the LC projection; and green arrows show the VLC projection.

The systematic arrangement between frequency and place was observed decades ago showing an orderly relationship between the cochlea and parts of the central auditory system (Rose et al., 1963; Boudreau and Tsuchitani, 1973). The IC has been a focus of such studies using physiological recording methods to establish place-frequency maps (Merzenich and Reid, 1974; FitzPatrick, 1975; Portfors and Roberts, 2014). The substrate for this frequency organization is initiated in the cochlea (Müller et al., 2005), established in the CN (Muniak et al., 2013), and passed along to the components of the SOC and higher centers. The distinct yet separate contributions to this organization in the IC is evident for the VCN and DCN (Figure 8). The projection pattern shows both convergence and divergence, a reminder of the involvement of frequency in processing multiple parameters of sound.

### Terminal endings

**FIGURE 9:**
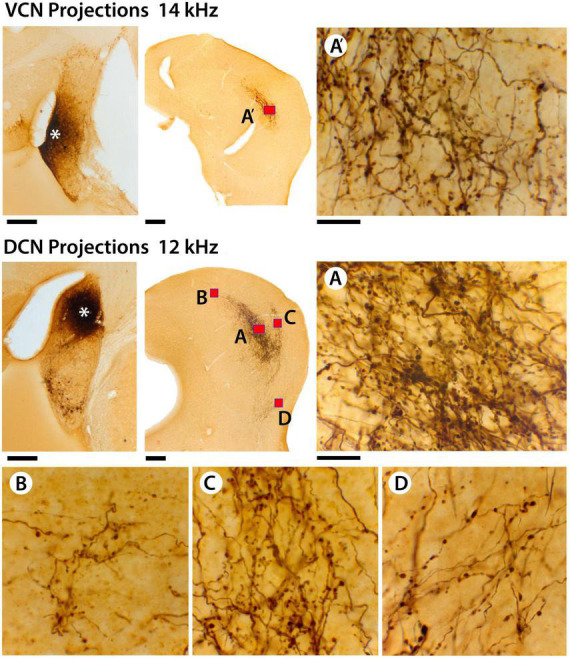
This figure provides higher magnification views of the differences and similarities between terminal fields from VCN and DCN projections. In the top row, there is the injection site in the VCN (far left, asterisk), the terminal field in the contralateral CNIC (red box, **A’**), and a high magnification photomicrograph (100× oil objective) through the middle of the terminal field **(A’)**. There are large and small terminals as well as thick and thin fibers. In the middle row, there is the DCN injection site (middle row left, asterisk) and the terminal fields in the contralateral IC (red boxes). The DCN terminal field has 4 components: **(A)** the CNIC; **(B)** the DC; **(C)** the LC; and **(D)** the VLIC. The distribution and pattern of terminals and fibers in the different zones are qualitatively similar with thick and thin fibers and large and small terminals. Quantitatively, there is no difference in the average size of terminals in the different subdivisions (see text). What seems to vary is the density of the projections. It should be noted, however, that the density or magnitude of the injections varies with respect to the individual animal, the injection parameters, and probably histological processing variables. Scale bars: injection sites and IC, 250 μm; high magnification images = 25 μm.

The size, shape, density, and distribution of the terminals from VCN and DCN projections into the IC were reliably stable. CN terminals in the IC could be qualitatively categorized as small or large by visual inspection (Figure 9). The pattern of axon branching and distribution of synaptic boutons appeared characteristic for each IC subdivision. The fibers were thick or thin and the endings were large or small. The thick fibers branched roughly every 100 μm, alternating with a long and a short branch. The short branches were 2–5 μm in length, terminating as a single bouton 3–4 μm in diameter. The thin fibers branched more frequently but irregularly; they also gave rise to *en passant* swellings, similar in size and shape as those arising from the thick fibers (3–4 μm in diameter). Secondary branches varied in length and exhibited large and small terminal and *en passant* swellings. While the terminal patterns were consistent within the different IC subdivisions, the branching density and number of terminals seemed related to the size of the injection site.

**FIGURE 10:**
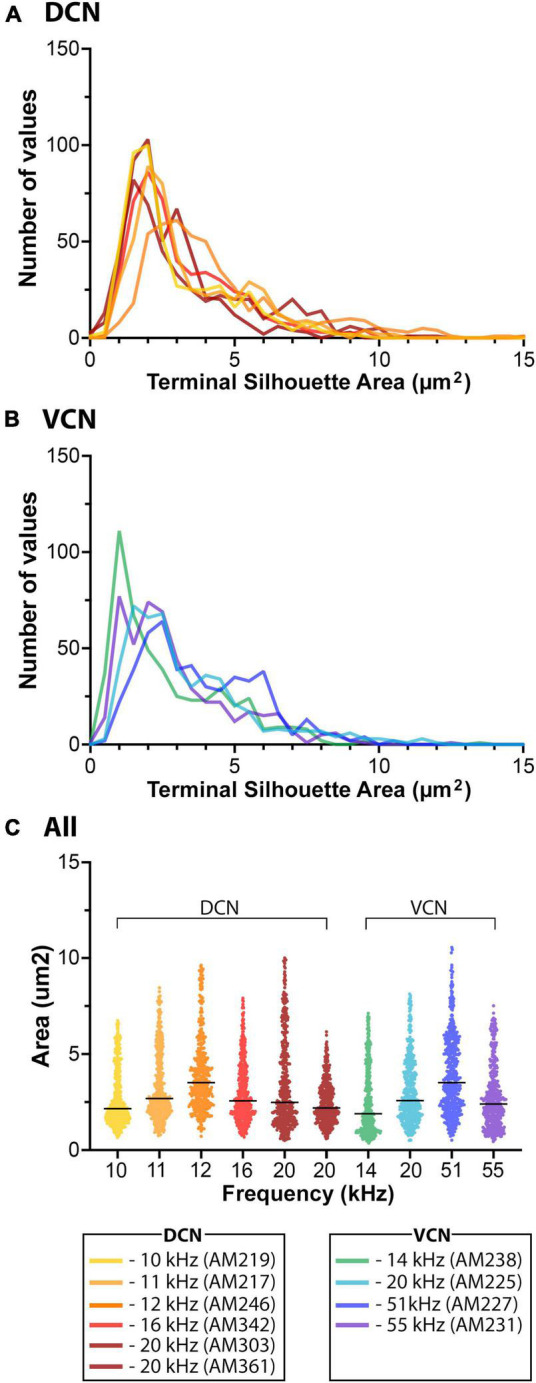
This histogram shows the size distribution of 485 randomly selected endings from each of 6 DCN (**A**, yellow-rust) and 4 VCN (**B**, green-purple) terminal fields in the CNIC. Despite the different frequency responses at the injection site, the terminals exhibit qualitatively similar patterns: a right skew with more small terminals than large ones. **(C)** The mean size of the terminal endings (silhouette area) did not differ when comparing those from the DCN versus those from the VCN (*p* = 0.914, two-tailed Mann–Whitney Test).

**FIGURE 11:**
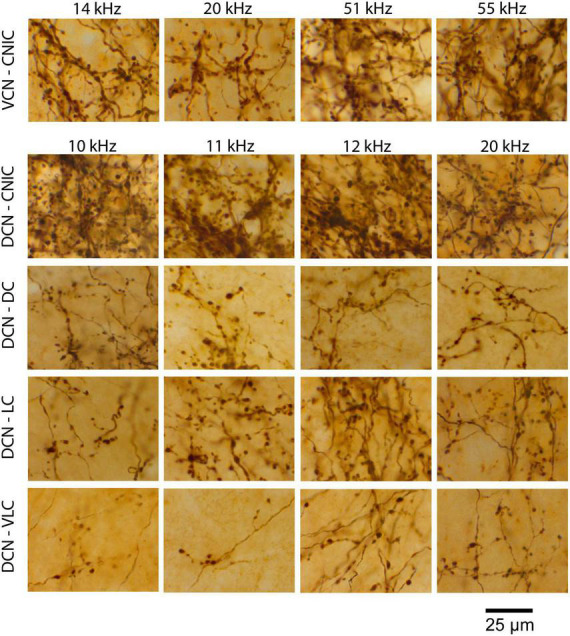
High magnification photomicrographs illustrating the qualitatively similar pattern of VCN and DCN fiber arborizations and terminal appearance for the separate IC subdivisions.

The average size of all terminals in the CNIC from the VCN and DCN projections (Figure 9, red boxes labeled A and A’) was not statistically different (VCN, 3.3.08 ± 0.62 μm^2^; DCN, 3.16 ± 0.54 μm^2^; Mann Whitney Test, 2-tailed, *p* = 0.914). The skewed distribution emphasized that there were many more small terminal endings compared to larger ones (Figure 10). When comparing the average size of terminals sampled from the IC (Figure 11, red boxes A, A’, B, C, D), there was no statistical difference in their means (CNIC, 2.60 ± 1.32 μm^2^; DCIC, 2.47 ± 1.51 μm^2^; LCIC, 2.46 ± 1.26 μm^2^; and VLC, 2.96 ± 1.81 μm^2^; Mann Whitney Test, 2 tailed, *p* = 0.12.

The pattern of terminations from VCN and DCN projections were qualitatively the same to the CNIC and DC: both projected strongly to the contralateral CNIC with some fibers extending into the DC. Very few fibers (0–3) were observed going to the ipsilateral IC in any mouse. In addition, however, the DCN projected contralaterally to the LC and VLC, with some fibers continuing to the ipsilateral medial geniculate nucleus. For the DCN, the CNIC was the main target with the LC a prominent secondary target; projections to the DC and VLC were smaller but present in all cases.

### Laminar versus paralaminar projections

**FIGURE 12:**
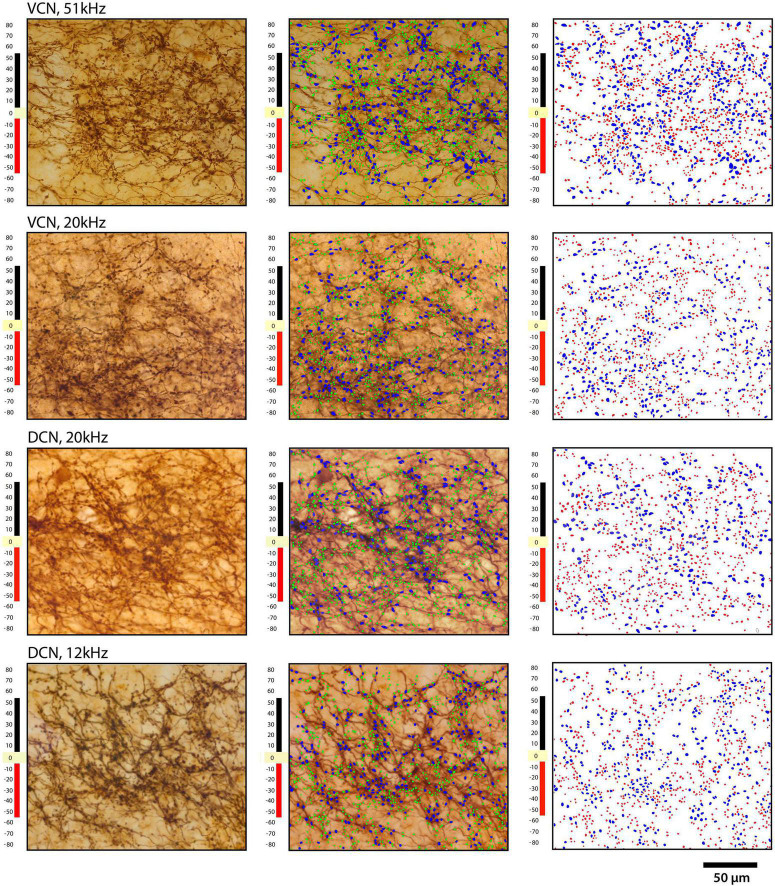
Plots of terminal distribution with respect to distance from laminar axis, set at zero. Each row shows the same area of IC from 2 VCN injections (top two rows) and two DCN injections: **left** column, photomicrograph through the middle of a projection lamina; **middle** column, photomicrograph with large and small endings drawn; **right** column shows only the drawings of terminals. Blue represents the large terminals, whereas red represents the small terminals. The ordinate illustrates the distance in micrometers from the central axis of the projection. Note the relative uniform distribution of large and small terminals.

**FIGURE 13:**
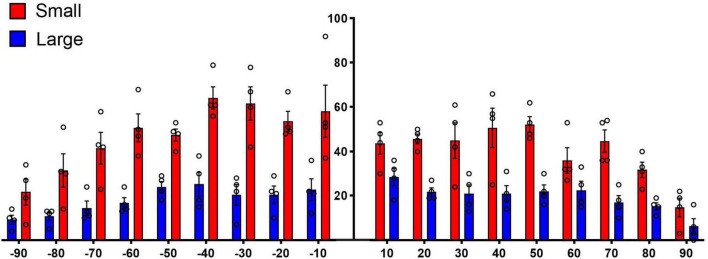
Plot of terminal distribution with respect to perpendicular distance from the laminar axis, data combined from measurements taken from Figure 12. All terminals within 10-μm-thick sectors were sorted into large or small categories and counted with respect to distance from the laminar axis (0). There is a slight but progressive decrease in the presence of large terminals associated with the distance from the central axis of a lamina (R squared = 0.5288, *p* = 0.04, two-tailed Pearson r) but not so with the small terminals (R squared = 0.1775, *p* = 0.30) or with all terminals combined (R squared = 0.3364, *p* = 0.13). These data do not show that the large terminals define the laminar axis and the small terminals are para-laminar as reported in cats and rats (Malmierca et al., 2005). There is the expected numerical fall-off on either side of the terminal axis (**right** and **left** ends of the X axis) for both large and small terminals.

It has been previously reported that projections from the CN were organized into a laminar and paralaminar pattern (Malmierca et al., 2005). In cats and rats, thick fibers and large terminal endings were distributed primarily in the middle of the terminal field that define the fibrodendritic and isofrequency lamina; in contrast, thin fibers and small terminals were distributed throughout the terminal field and noticeably also along the edges of the terminal field where large endings were absent. In our CBA/CaH mice, however, we did not observe this pattern. Large and small terminal endings appeared uniformly mixed within the CNIC terminal fields of VCN and DCN projections (Figure 12). This distribution was quantified by parsing the terminal field into parallel strips, 10 μm wide, extending away from the central axis of the isofrequency field (Figure 12, labeled 0). The number of large and small endings in each strip was plotted with respect to the distance from the central axis of the lamina. Although there were many more small endings than large endings, counts of each type co-varied together according to position with endings densest near the central axis of the lamina and declining gradually and progressively toward the edge of the terminal field (Figure 13).

## Discussion

The present report contributes new data on the fundamental organization of circuitry in the ascending auditory system. The application of electrophysiological recordings and anterograde tracing methods complement what we know from retrograde pathway studies and broaden the comparative foundation for studying the structural biology of hearing reported for cats, guinea pigs, rats, and gerbils. A summary of our findings in the mouse is as follows: (1) the DCN and VCN have distinctly separate projections to the contralateral IC as determined using anterograde tracing methods and injections into electrophysiologically defined regions - VCN projections to the IC terminate primarily to the CNIC, whereas DCN projections to the IC terminate in the CNIC, DC, LC and VLC; (2) the projection sheets are topographic and tonotopic where a frequency field is contained within the subjacent higher frequency fields; (3) fibers originating from the VCN travel in a lateral position within the LL, whereas those of the DCN travel in a medial position irrespective of frequency; and (4) VCN and DCN projections give rise to large and small endings that appear uniformly distributed within their respective terminal field.

### CN projections

**FIGURE 14:**
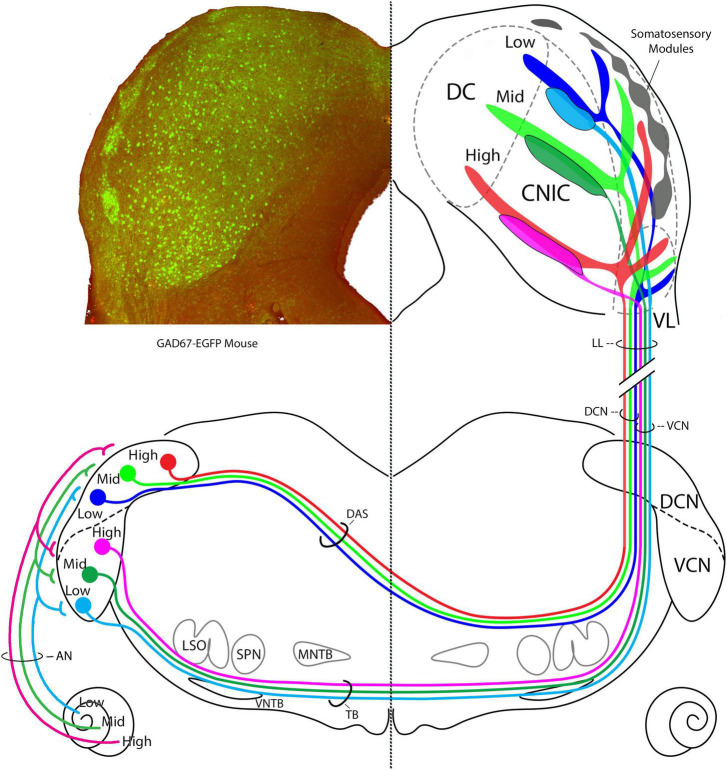
Summary diagram of CN projections to the IC. **Left** IC is taken from a transgenic GAD67-EGFP mouse and shows layer II modules of the lateral cortex, site of somatosensory input. GAD67 positive cell bodies (green) are presumed GABAergic and are also revealed in the CNIC and DC. The AN has tonotopic projection to the VCN and DCN (Müller et al., 2005; Muniak et al., 2013). In the **right** IC, the terminal fields of projections from the AVCN and the DCN are schematized. AVCN projections terminate almost exclusively in the CNIC, whereas DCN projections terminate in the CNIC, DC, layer III of the LC, and the VL region of the LC. We could not determine if the terminal frequencies having the same frequencies are congruent. The DCN and AVCN axons are segregated in the lateral lemniscus into medial and lateral positions, respectively, before terminating in a topographic way in the IC. These auditory inputs do not appear to mix with somatosensory inputs in the LC modules. How the modules and auditory projections interact will be key to understanding multimodal processing at this level.

We summarize the VCN and DCN projection patterns to the IC as revealed using anterograde tracers in the CBA/CaH mouse (Figure 14). It has been long known that these structures project to the IC. Retrograde labeling following relatively large dye injections into the IC provided further answers as to what regions (Brunso-Bechtold et al., 1981; Frisina et al., 1998) and what types of neurons were projecting (Beyerl, 1978; Adams, 1979; Cant, 1982). Our anterograde projection data complement retrograde evidence by showing the differential terminations by the VCN and DCN in the IC (Ryugo et al., 1981).

The IC is a hub for ascending and descending auditory pathways with proposed subdivisions serving as informational switchboards (Winer et al., 2005). The VCN with its confined connection to the CNIC implies a purely auditory role in the perception of sound. The CNIC contains neurons with tufted dendrites (Rockel and Jones, 1973, Morest and Oliver, 1984) and sharp frequency tuning (Aitkin et al., 1975; Portfors et al., 2011), consistent with the hypothesized, modality-specific “lemniscal” pathways of the brain (Lorente de Nó, 1938; Ramon-Moliner, 1962; Graybiel, 1973). In contrast, the DCN contributes to both a lemniscal and non-lemniscal system. LCIC is a multimodal convergence zone (Robards, 1979; Bácskai et al., 2002; Dillingham et al., 2017; Liu et al., 2023) with physiological response properties that seem to deal more with the physical context of sound (Aitkin et al., 1978). The different CN projections to the IC have a practical implication: the effectiveness of auditory brainstem implants in the CN as reflected by IC responses favors the VCN over the DCN as the preferred target for stimulation (McInturff et al., 2022, 2023).

Type I stellate cells in the core of the VCN and small stellate cells in the VCN margins project to the IC (Roth et al., 1978; Adams, 1979; Cant, 1982; Doucet and Ryugo, 2006). Data from the cat and rat yielded similar but not identical conclusions about CN projections in mice. For instance, single injections into physiologically defined regions of the DCN and/or VCN labeled two prominent bands of terminals; a main band in the CNIC and a shorter lateral band in the LC (Malmierca et al., 2005; Loftus et al., 2008). This dual terminal field is distinctly absent from the VCN projections of mice (Figures 3, 8). Moreover, in an exhaustive study of the gerbil (Cant and Benson, 2006), cells in the VCN, PVCN and DCN were labeled in 72 of the 74 injections into various parts of the IC. We cannot explain these differences, but the most plausible interpretation involves species variations and their different natural histories. With respect to cats and rats, mice occupy a different position in the food chain—mice are prey for cats and rats (Yang et al., 2004; Campos et al., 2013; Lahger and Laska, 2018). Gerbils are social, desert animals that live in multibranched burrows (Ågren, 1976; Scheibler et al., 2005), whereas mice inhabit forests, grasslands, or urban areas and live in simple burrows (Fisher and Llewellyn, 1978; Bradford, 2014). It is argued that different lifestyles exerted selective survival pressures on certain brain features of individual species. In spite of these variations, it should be stressed that the fundamental design of the central auditory system is similar across mammals.

Natural selection requires adaptation of different animals to their habitat, so brain specializations and variations should be expected. The differences in hearing range (gerbils, 0.1–60 kHz; mice, 4–90 kHz) and structure of certain auditory brainstem nuclei offer potential clues into what features of the sound stimulus are providing information necessary for predator avoidance and social communication (Fay, 1988). Both animals have small heads, which limit the utility of head shadow effects and impose physical limits on interaural time differences (Grothe, 2000). The task, then, is to collect neural evidence to explain how interaural disparity cues are transformed into spatial awareness by the separate pathways.

The majority of pathway tracing studies have used retrograde tracing methods (Cant and Benson, 2003, 2006). Even when an injection of a retrograde tracer is placed precisely on target, the dye marking the injection site will obscure important details about the anatomy of the projection, such as the branching structure of individual fibers, the organizational pattern of terminals, and the target neurons. Thus, the use of anterograde tracing methods provides data that are independent yet complementary to those revealed by retrograde studies. When applying pathway tracing methods, it is also important to be aware of technical differences and limitations (Mesulam et al., 1980; Saleeba et al., 2019, 2020), the impact of age (LeVay et al., 1980; Ryugo and Fekete, 1982; Willott, 1984; Frisina and Walton, 2001), and the importance of species (Irving and Harrison, 1967; Moore and Moore, 1971; Merzenich et al., 1973; Masterton et al., 1975). These variables have an important role in the interpretation of the results.

Pyramidal and giant cells are sources of DCN input to the IC (Osen, 1972; Beyerl, 1978; Ryugo et al., 1981; Oliver, 1984; Ryugo and Willard, 1985). These cells exhibit narrow frequency tuning (Godfrey et al., 1975; Rhode et al., 1983; Rhode and Smith, 1986) but they differ in terms of their dendritic orientation and cell body location in the DCN (Brawer et al., 1974; Schweitzer and Cant, 1985). Pyramidal cells are the dominant neuron, located in layer II and exhibiting a planar dendritic field that form frequency layers across the long axis of the nucleus (Blackstad et al., 1984; Ryugo and Willard, 1985; Spirou et al., 1993). The dendritic structure of pyramidal cells, aligned with the incoming auditory nerve fibers, is consistent with their sharp tuning. In contrast, giant cells represent a much smaller numerical population, are located in the deep layers of the DCN and exhibit widely branching dendrites, consistent with their intercepting of a variety of diverse inputs (Ryugo and Willard, 1985). Because both cells have sharp tuning, the planar dendrites of pyramidal cells intercept a narrow frequency band of input. The giant cells, with their wide dendritic branching, might receive frequency-specific auditory input exclusively onto their cell bodies but accept diverse inputs onto their dendrites. In this way, both cell types could exhibit relatively narrow tuning and frequency-specific projections but the giant cells would be capable of conveying a richer palate of information further upstream.

### Tonotopy

We noted three tonotopic projection arms into the IC from the DCN. There were the previously reported main CNIC projection (that included a lateral part of the DC) and a second projection arm into layer III of the LC, plus the newly detected short projection branch into the VLC. The two main branches were inferred to endow the IC with its tonotopic organization, where the frequency axes of the two projection arms were orthogonal to each other yet conformed to the frequency features recorded in cats (Aitkin et al., 1975; Loftus et al., 2008).

The three branches of the DCN projection were distinct and present in all our mice. The CN projections in cat and rat were shown to stack (Loftus et al., 2008), a relationship verified by us for the mouse. When sections at the level the IC commissure are selected from the different mice, superimposed, and scaled, the result is an imperfect but credible stacking of DCN frequency projections. This tonotopic stacking, where one frequency band “nests” into the one below, implies that there is a proportional relationship to frequency bands, with the largest band resting at the ventral edge of the CNIC, and progressively smaller bands stacked up to the dorsal extremity. Moreover, this stacking includes the three arms of the DCN input to the IC (Figure 14). This relationship implies that there is a gradual but progressive increase in tissue devoted to processing higher frequency sounds.

The apparent expansion of tissue representing higher frequencies is noteworthy because the highest frequency response we’ve recorded in the mouse auditory brainstem is 56 kHz, whereas the hearing range of the mouse extends beyond 100 kHz. Our lab data are consistent with the results of Portfors and Roberts (2014) who suggest that the mid-high frequency regions of the IC are processing the lower frequency harmonics of high frequencies. Such an adaptation would be a clever way of accessing high frequency auditory stimuli without having to devote new brain tissue for the expanded sensitivity.

The tuning characteristics of IC units in the three IC divisions have been described; all units exhibited frequency tuning curves, but DC and LC units were more broadly tuned and “habituated” after a few stimulus presentations (Aitkin et al., 1975). Moreover, in recordings free of anesthesia effects, IC responses were shown to resemble those of the DCN, excited by low levels of stimulation and silenced by higher levels (Davis et al., 2003); these neurons were also sensitive to spectral troughs created by pinna cues (Rice et al., 1992). The spectral changes caused by head-related transfer functions means that a particular sound, composed of its own unique spectrum of frequencies, will change depending on its location in space by virtue of its altered spectrum. Synthesized waveforms played through headphones that simulate pinna transformations mimicked free-field listening and generally permitted source location in space (Wightman and Kistler, 1989a,b). For sounds to maintain their identity when subject to wide ranging variations in spectral waveforms depending on environmental conditions and/or source location, there must be real-time, ongoing auditory connections to memory circuits.

### Terminals

The terminal endings of the CN projections are, in a simplistic way, large or small. There are an estimated 2–3× as many small endings as large ones, and the two ending types appear uniformly distributed within the terminal fields. We tested the hypothesis, proposed by Malmierca et al. (2005), that the large terminals would define the laminar axis, whereas the small terminals would lie on the “paralaminar” periphery (Supplementary Figure 5). The seemingly uniform mixture of large and small terminals over the terminal field for the CBA/CaH mouse is not consistent with that plan. Moreover, we did not observe large endings arising from thick fibers and small endings arising from thin fibers; rather, endings of either size could arise of fibers of either thickness. Fiber thickness is not always a reliable anatomical feature because myelinated fibers often appear thinner as they pass through the depth of the tissue section. This artifact was frequently observed for myelinated auditory nerve fibers and was attributed to inadequate penetration of the chromogenic reagents through the tissue and especially the myelin (Fekete et al., 1984). The endings in our material were overly stained so no internal details of the endings were visible when viewed in an electron microscope. It was evident, however, that the labeled endings from the CN were apposed entirely against dendrites in the CNIC, in agreement with what was observed in cats (Oliver, 1985).

### Convergence of multimodal inputs

Pyramidal and perhaps giant cells are also innervated at the apical ends of their dendrites by parallel fibers of granule cells (Mugnaini et al., 1980a,b; Manis, 1989; Shore and Moore, 1998; Rubio et al., 2008). Granule cells are known for their integration of multimodal inputs (Shore and Moore, 1998; Ryugo et al., 2003). These inputs include projections from the somatosensory system that would provide information from proprioceptors from the head, neck, and pinna (Robards, 1979; Weinberg and Rustioni, 1987; Wright and Ryugo, 1996; Haenggeli et al., 2005; Zhou and Shore, 2006). Information from the vestibular system would inform the animal about head and spine position with respect to gravity, movement, posture, and 3D space (Künzle, 1973; Flumerfelt et al., 1982; Shokunbi et al., 1985; Walberg et al., 1985; Burian and Gstoettner, 1988; Kevetter and Perachio, 1989; Bácskai et al., 2002; Bukowska, 2002; Newlands and Perachio, 2003). Such information is interacting with sound processing early in the ascending auditory pathway (Kanold and Young, 2001; Kanold et al., 2011). Multimodal information from the cerebral cortex by way of the pons (Ohlrogge et al., 2001), from the cerebellum via the lateral reticular nucleus (Zhan and Ryugo, 2007), and from the visual system (Qvist and Dietrichs, 1985, 1986; McCrea et al., 1987) is also being delivered. The output of the DCN has already been subject to sophisticated processing before it gets delivered to the IC (Young et al., 1992; Nelken and Young, 1996; Davis et al., 2003). The IC exhibits both convergence and compartmentalization of its diverse inputs (Lamb-Echegaray et al., 2019; Weakley et al., 2022) so we are reminded that navigating in our acoustic environment requires more than just the transduction of vibrations in air.

Our cognitive perception of the world is richly multimodal. Stereognosis, the ability to identify the 3D shape of an object manually or orally is linked to auditory discrimination (Lewis and Kelly, 1974). The ventriloquism effect demonstrates that sound localization is influenced by vision (Spence and Driver, 2000), and facial movements can affect the perception of speech (McGurk and MacDonald, 1976) as well as non-speech stimuli (Saldaña and Rosenblum, 1993; Laeng et al., 2021). There is also the sound-induced flash illusion where the observer misinterprets the number of visual flashes due to the simultaneous presentation of a different number of clicks (Virsu et al., 2008). Audiotactile interactions are dramatically illustrated by the parchment-skin illusion where tactile sensation is influenced by sound (Jousmäki and Hari, 1998) in sighted but not blind individuals (Champoux et al., 2011). These illusions or misinterpretations of our sensory experience nonetheless depend on the essential parameters of sound – frequency, timing and experience. No illusion would occur if the sound of a trumpet came from a moving mouth or if speech was produced by rubbing hands together.

Species differences, habitat ecology, and hearing behavior provide insight for understanding circuits. The VCN and the DCN exhibit differential projections to the IC. The idea of parallel processing is a little misleading because ascending circuits convey different kinds of information and synaptic mechanisms at the targets are variable. The VCN primarily deals with frequency and timing of auditory information, whereas the DCN integrates auditory and non-auditory inputs. They both project to the IC while maintaining separate domains. Multimodal integration at the levels of the DCN and lateral cortex of the IC executes processes that help integrate sound and context for sorting concurrent sound streams. These and other circuits are presumed to enable sound recognition under a variety of different conditions, where the spectral composition changes but our recognition does not. It is anticipated that identifying species-differences in auditory circuits will contribute to a better understanding of central mechanisms of hearing.

## Data availability statement

The raw data supporting the conclusions of this article will be made available by the authors, without undue reservation.

## Ethics statement

The animal study was reviewed and approved by the Animal Ethics Committee of the Garvan Institute and St. Vincent’s Hospital, UNSW Australia. The work was performed in strict accordance with the Australian Code for the Care and Use of Animals for Scientific Purposes (2013) and the ethical guidelines of the National Health and Medical Research Council (NHMRC) of Australia. All animals were handled according to the Animal Ethics Committee protocols (Animal Research Authority: 14-01, 14-03, 16-28, 19-33, 20-02, and 21-13).

## Author contributions

GM conducted the recordings, injections, histological processing, and all the fluorescent photomicroscopy under the supervision of DR. DR performed the initial analysis of the data, prepared the figures, composed the first draft of the manuscript, and secured funding for the project. Both authors designed the research and verified the accuracy and integrity of the work as well as contributed to the final draft of the manuscript and figures.

## Supplementary material

Supplementary Figure 1 Diagram of the main subdivisions of the mouse IC. The divisions follow the criteria established in an array of publications (Ryugo et al., 1981; Faye-Lund and Osen, 1985; Stebbings et al., 2014; Lesicko et al., 2016; Milinkeviciute et al., 2017; Weakley et al., 2022). The somatosensory modules (purple ovals, left image) are shown in layer 2 of the LC (middle image). The right image shows the traced fibers from the DCN (black) and VCN (blue) with the subdivisions.

Supplementary Figure 2 This series of images review the process of fiber tracing for improving visualization of terminal patterns at low magnification. (Panel 1) Photographic montage of an IC section collected at 800 dpi using a 10× objective compared to a montage (Panel 2) of the same section collected using a 40× objective. (Panels 3–4) Each image was then digitally magnified so that labeled fibers and terminals in the IC could be manually drawn using a drawing tablet. We compared image collected with the 10× objective (Panel 5) to image collected using the 40× objective (Panel 6) and determined that qualitatively, there was not a major difference in terminal field plots when tracing the fibers photographed using a 10× or 40× objective (compare Panel 7 to Panel 8). These comparisons confirmed the utility of digitized magnification, the adequacy of 10× photomontages for terminal field plotting, and significantly reduced the amount of time needed to collect images for analysis.

Supplementary Figure 3 This series of images illustrate how terminal fields were enhanced for low magnification viewing. Photographic tile sets that encompassed the entire IC across serial sections were collected using 10× objectives and were manually montaged (Panel 1) and then digitally magnified (Panel 2). The same field was photographed using a 20× objective (Panel 3). The digitized image was resampled at 800 dpi and then magnified again so that individually labeled fibers and terminals in the IC could be manually drawn (Panel 4). The drawings and the photomicrograph were merged (Panel 5), scaled, and repositioned back onto the photomontage made with the 10× objective (Panel 6). This procedure brought out details that were not apparent prior to the tracing (compare Panel 1 to Panel 6). This method enhanced the contrast and details of terminal field patterns. Scale bars: 250 μm for panels 1 and 6; 100 μm for panels 2–5.

Supplementary Figure 4 Our digital tracing method was used to compare projection patterns from chromogenic and fluorescent material. In the top row (1), we show how tracing improves visualizing in brightfield images; in the bottom row (2), we show a similar improvement in fluorescent images. This method is excellent for showing “global” patterns.

Supplementary Figure 5 This figure shows how we analyzed the distribution of terminal endings with respect to the pattern of laminar projections. **(A)** The DCN projections to the IC are demonstrated by the darkly stained fibers and terminals revealed by a chromogenic reaction using diaminobenzidine. The blue arrows flank the curved lamina and the red rectangle shows the sampling area. **(B)** The red box in panel **(A)** is shown where 0 approximates the middle of the lamina and represents the laminar “axis.” The horizontal lines are 10 μm apart. A visual assessment of terminal distribution is confirmed by counts, where the number of large and small terminals covaries together in each sector. The laminar axis is not highlighted by a preferential concentration of large terminals. Terminal ending density does diminish at the edges of the terminal field.

Supplementary Table 1 Summary table of all DCN and AVCN injections used in the study.

## References

[B1] Adams J. C. (1979). Ascending projections to the inferior colliculus. *J. Comp. Neurol.* 183 519–538. 10.1002/cne.901830305 759446

[B2] Adams J. C. Warr W. B. (1976). Origins of axons in the cat’s acoustic striae determined by injection of horseradish peroxidase into severed tracts. *J. Comp. Neurol.* 170 107–122. 10.1002/cne.901700108 61976

[B3] Ågren G. (1976). Social and territorial behaviour in the mongolian gerbil (*Meriones unguiculatus*) under seminatural conditions. *Biol. Behav.* 1 267–285.

[B4] Aitkin L. M. Dickhaus H. Schult W. Zimmermann M. (1978). External nucleus of inferior colliculus: Auditory and spinal somatosensory afferents and their interactions. *J. Neurophysiol.* 41 837–847. 10.1152/jn.1978.41.4.837 681989

[B5] Aitkin L. M. Kenyon C. E. Philpott P. (1981). The representation of the auditory and somatosensory systems in the external nucleus of the cat inferior colliculus. *J. Comp. Neurol.* 196 25–40. 10.1002/cne.901960104 7204665

[B6] Aitkin L. M. Webster W. R. Veale J. L. Crosby D. C. (1975). Inferior Colliculus. I. Comparison of response properties of neurons in central, pericentral, and external nuclei of adult cat. *J. Neurophysiol.* 38 1196–1207. 10.1152/jn.1975.38.5.1196 1177012

[B7] Bácskai T. Székely G. Matesz C. (2002). Ascending and descending projections of the lateral vestibular nucleus in the rat. *Acta Biol. Hung.* 53 7–21. 10.1556/abiol.53.2002.1-2.3 12064781

[B8] Bajo V. M. King A. J. (2013). Cortical modulation of auditory processing in the midbrain. *Front. Neural Circuits* 6:114. 10.3389/fncir.2012.00114 23316140 PMC3539853

[B9] Beyerl B. D. (1978). Afferent projections to the central nucleus of the inferior colliculus in the rat. *Brain Res.* 145 209–223. 10.1016/0006-8993(78)90858-2 638786

[B10] Björkeland M. Boivie J. (1984). An anatomical study of the projections from the dorsal column nuclei to the midbrain in cat. *Anat. Embryol.* 170 29–43. 10.1007/BF00319455 6089608

[B11] Blackstad T. W. Osen K. K. Mugnaini E. (1984). Pyramidal neurones of the dorsal cochlear nucleus: A Golgi and computer reconstruction study in cat. *Neuroscience* 13 827–854. 10.1016/0306-4522(84)90099-x 6527780

[B12] Boudreau J. Tsuchitani C. (1970). Cat superior olive S-segment cell discharge to tonal stimulation. *Contribut. Sens. Physiol.* 4 143–213. 10.1016/b978-0-12-151804-2.50011-5 4914058

[B13] Boudreau J. Tsuchitani C. (1973). *) The Auditory System. Sensory Neurophysiology.* New York, NY: Van Nostrand Reinhold Co.

[B14] Bradford A. (2014). *Mouse facts: habits, habitat and types of mice.* Lehi, UT: Life and Science.

[B15] Brawer J. R. Morest D. K. Kane E. C. (1974). The neuronal architecture of the cochlear nucleus of the cat. *J. Comp. Neurol.* 155 251–300. 10.1002/cne.901550302 4134212

[B16] Brunso-Bechtold J. K. Thompson G. C. Masterton R. B. (1981). HRP study of the organization of auditory afferents ascending to central nucleus of inferior colliculus in cat. *J. Comp. Neurol.* 197 705–722. 10.1002/cne.901970410 7229134

[B17] Bukowska D. (2002). Morphological evidence for secondary vestibular afferent connections to the dorsal cochlear nucleus in the rabbit. *Cells Tissues Organs* 170 61–68. 10.1159/000047921 11602803

[B18] Burian M. Gstoettner W. (1988). Projection of primary vestibular afferent fibres to the cochlear nucleus in the Guinea pig. *Neurosci. Lett.* 84 13–17. 10.1016/0304-3940(88)90329-1 2831482

[B19] Campos K. F. C. Amaral V. C. S. Rico J. L. Miguel T. T. Nunes-de-Souza R. L. (2013). Ethopharmacological evaluation of the rat exposure test: A prey-predator interaction test. *Behav. Brain Res.* 240 160–170. 10.1016/j.bbr.2012.11.023 23195112

[B20] Cant N. B. (1982). Identification of cell types in the anteroventral cochlear nucleus that project to the inferior colliculus. *Neurosci. Lett.* 32 241–246. 10.1016/0304-3940(82)90300-7 7177487

[B21] Cant N. B. Benson C. G. (2003). Parallel auditory pathways: Projection patterns of the different neuronal populations in the dorsal and ventral cochlear nuclei. *Brain Res. Bull.* 60 457–474. 10.1016/s0361-9230(03)00050-9 12787867

[B22] Cant N. B. Benson C. G. (2006). Organization of the inferior colliculus of the gerbil (meriones unguiculatus): Differences in distribution of projections from the cochlear nuclei and the superior olivary complex. *J. Comp. Neurol.* 495 511–528. 10.1002/cne.20888 16498677 PMC2566545

[B23] Cant N. B. Benson C. G. (2008). Wisteria floribunda lectin is associated with specific cell types in the ventral cochlear nucleus of the gerbil, meriones unguiculatus. *Hear. Res.* 21 64–72. 10.1016/j.heares.2006.01.008 16497454

[B24] Carpenter M. B. (1978). *Core Text of Neuroanatomy*, 2nd Edn. Baltimore: Williams and Wilkins.

[B25] Champoux F. Collignon O. Bacon B. A. Lepore F. Zatorre R. J. Théoret H. (2011). Early- and late-onset blindness both curb audiotactile integration on the parchment-skin illusion. *Psychol. Sci*. 1 19–25. 10.1177/0956797610391099 21123856

[B26] Coleman J. R. Clerici W. J. (1987). Sources of projections to subdivisions of the inferior colliculus in the rat. *J. Comp. Neurol.* 262 215–226. 10.1002/cne.902620204 3624552

[B27] Connelly C. J. Ryugo D. K. Muniak M. A. (2017). The effect of progressive hearing loss on the morphology of endbulbs of held and Bushy Cells. *Hear. Res.* 343 14–33. 10.1016/j.heares.2016.07.004 27473502

[B28] Darrow K. N. Benson T. E. Brown M. C. (2012). Planar multipolar cells in the cochlear nucleus project to medial olivocochlear neurons in mouse. *J. Comp. Neurol.* 520 1365–1375. 10.1002/cne.22797 22101968 PMC3514887

[B29] Davis K. A. Ramachandran R. May B. J. (2003). Auditory processing of spectral cues for sound localization in the inferior colliculus. *J. Assoc. Res. Otolaryngol.* 4 148–163. 10.1007/s10162-002-2002-5 12943370 PMC3202719

[B30] Dillingham C. H. Gay S. M. Behrooz R. Gabriele M. L. (2017). Modular-extramodular organization in developing multisensory shell regions of the mouse inferior colliculus. *J. Comp. Neurol.* 525 3742–3756. 10.1002/cne.24300 28786102 PMC5832046

[B31] Doucet J. R. Ross A. T. Gillespie M. B. Ryugo D. K. (1999). Glycine immunoreactivity of multipolar neurons in the ventral cochlear nucleus which project to the dorsal cochlear nucleus. *J. Comp. Neurol.* 408 515–531. 10340502

[B32] Doucet J. R. Ryugo D. K. (1997). Projections from the ventral cochlear nucleus to the dorsal cochlear nucleus in rats. *J. Comp. Neurol.* 385 245–264. 9268126

[B33] Doucet J. R. Ryugo D. K. (2003). Axonal pathways to the lateral superior olive labeled with biotinylated dextran amine injections into the dorsal cochlear nucleus of rats. *J. Comp. Neurol.* 461 452–465. 10.1002/cne.10722 12746862

[B34] Doucet J. R. Ryugo D. K. (2006). Structural and functional classes of multipolar cells in the ventral cochlear nucleus. *Anat. Rec. Part A* 288A 331–344. 10.1002/ar.a.20294 16550550 PMC2566305

[B35] Evans E. F. Nelson P. G. (1973). The responses of single neurons in the cochlear nucleus of the cat as a function of their location and the anesthetic state. *Exp. Brain Res.* 14 402–427. 10.1007/BF00234103 4725899

[B36] Fay R. R. (1988). *Hearing in Vertebrates: A Psychophysics Databook.* Winnetka, IL: Hill-Fay Associates.

[B37] Faye-Lund H. Osen K. K. (1985). Anatomy of the inferior colliculus in rat. *Anat. Embryol.* 171 1–20. 10.1007/bf00319050 3985354

[B38] Fekete D. M. Rouiller E. M. Liberman M. C. Ryugo D. K. (1984). The central projections of intracellularly labeled auditory nerve fibers in adult cats. *J. Comp. Neurol.* 229 432–450. 10.1002/cne.902290311 6209306

[B39] Fisher M. F. Llewellyn G. C. (1978). The Mongolian gerbil: Natural history, care, and maintenance. *Am. Biol. Teach.* 40 557–560. 10.2307/4446413

[B40] FitzPatrick K. A. (1975). Cellular architecture and tonpographic organization of the inferior colliculus of the squirrel monkey. *J. Comp. Neurol.* 164 185–208. 10.1002/cne.901640204 810498

[B41] Flumerfelt B. A. Hrycyshyn A. W. Kapogianis E. M. (1982). Spinal projections to the lateral reticular nucleus in the rat. *Anat. Embryol.* 165 345–359. 10.1007/bf00305572 7158817

[B42] Frisina R. Walton J. (2001). “Aging of the Mouse Central Auditory System,” in *Handbook of Mouse Auditory Research*, ed. Willott J. F. (Boca Raton, FL: CRC Press), 10.1201/9781420038736.ch24

[B43] Frisina R. D. Walton J. P. Lynch-Armour M. A. Byrd J. D. (1998). Inputs to a physiologically characterized region of the inferior colliculus of the young adult CBA Mouse. *Hear. Res.* 115 61–81. 10.1016/s0378-5955(97)00176-7 9472736

[B44] Godfrey D. A. Kiang N. Y.-S. Norris B. E. (1975). Single unit activity in the posteroventral cochlear nucleus of the cat. *J. Comp. Neurol.* 162 247–268. 10.1002/cne.901620206 1150921

[B45] Goldberg J. M. Brown P. B. (1969). Response of binaural neurons of dog superior olivary complex to dichotic tonal stimuli: Some physiological mechanisms of sound localization. *J. Neurophysiol.* 32 613–636. 10.1152/jn.1969.32.4.613 5810617

[B46] Gómez-Álvarez M. Saldaña E. (2016). different tonotopic regions of the lateral superior olive receive a similar combinationof afferent inputs. *J. Comp. Neurol.* 524 2230–2250. 10.1002/cne.23942 26659473

[B47] Graybiel A. M. (1973). The thalamo-cortical projection of the so-called posterior nuclear group: a study with anterograde degeneration methods in the cat. *Brain Res.* 49 229–244. 10.1016/0006-8993(73)90420-4 4720788

[B48] Grothe B. (2000). The evolution of temporal processing in the medial superior olive, an auditory brainstem structure. *Prog. Neurobiol.* 61 581–610. 10.1016/s0301-0082(99)00068-4 10775798

[B49] Grothe B. Pecka M. McAlpine D. (2010). Mechanisms of sound localization in mammals. *Physiol. Rev.* 90 983–1012. 10.1152/physrev.00026.2009 20664077

[B50] Guinan J. J. Guinan S. S. Norris B. E. (1972). Single auditory units in the superior olivary complex: I: Responses to sounds and classifications based on physiological properties. *Int. J. Neurosci.* 4 101–120. 10.3109/00207457209147165

[B51] Haenggeli C.-A. Pongstaporn T. Doucet J. R. Ryugo D. K. (2005). Projections from the spinal trigeminal nucleus to the cochlear nucleus in the rat. *J. Comp. Neurol.* 484 191–205. 10.1002/cne.20466 15736230

[B52] Haskell G. D. (2022). *Sounds Wild and Broken*. New York, NY: Viking.

[B53] Henry K. R. Chole R. A. (1980). Genotypic differences in behavioral, physiological and anatomical expressions of age-related hearing loss in the laboratory mouse. *Audiology* 19 369–383. 10.3109/00206098009070071 7436856

[B54] Huffman R. F. Henson O. W. (1990). The descending auditory pathway and acousticomotor systems: Connections with the inferior Colliculus. *Brain Res. Rev.* 15 295–323. 10.1016/0165-0173(90)90005-9 2289088

[B55] Irving R. Harrison J. M. (1967). The superior olivary complex and audition: A compartive study. *J. Comp. Neurol.* 130 77–86. 10.1002/cne.901300105 4962091

[B56] Itoh K. Kamiya H. Mitani A. Yasui Y. Takada M. Nizuno N. (1987). Direct projections from the dorsal column nuclei and the spinal trigeminal nuclei to the cochlear nucleiu in the cat. *Brain Res.* 400 145–150. 10.1016/0006-8993(87)90662-7 2434184

[B57] Jeffress L. A. (1948). A place theory of sound localization. *J. Comp. Physiol. Psychol.* 41 35–39. 10.1037/h0061495 18904764

[B58] Jousmäki V. Hari R. (1998). Parchment-skin illusion: sound-biased trouch. *Curr. Biol.* 8 R190–R191. 10.1016/s0960-9822(98)70120-4 9512426

[B59] Kanold P. O. Davis K. A. Young E. D. (2011). Somatosensory context alters auditory responses in the cochlear nucleus. *J. Neurophysiol.* 105 1063–1070. 10.1152/jn.00807.2010 21178001 PMC3295206

[B60] Kanold P. O. Young E. D. (2001). Proprioceptive information from the pinna provides somatosensory input to cat dorsal cochlear nucleus. *J. Neurosci.* 21 7848–7858. 10.1523/jneurosci.21-19-07848.2001 11567076 PMC6762891

[B61] Kevetter G. A. Perachio A. A. (1989). Projections from the sacculus to the cochlear nuclei in the Mongolian gerbil. *Brain Behav. Evol.* 34 193–200. 10.1159/000116505 2590835

[B62] Konishi M. (2000). Study of sound localization by owls and its relevance to humans. *Comp. Biochem. Physiol. Part A Mol. Integr. Physiol.* 126 459–469. 10.1016/s1095-6433(00)00232-4 10989338

[B63] Künzle H. (1973). The Topographic Organization of spinal afferents to the lateral reticular nucleus of the cat. *J. Comp. Neurol.* 149 103–115. 10.1002/cne.901490107 4700511

[B64] Laeng B. Kuyateh S. Kelkar T. (2021). Substituting facial movements in singers changes the sounds of musical intervals. *Sci. Rep.* 11:22442. 10.1038/s41598-021-01797-z 34789775 PMC8599708

[B65] Lahger C. Laska M. (2018). Behavioral responses of CD-1 mice to conspecific and heterospecific blood odors and to a blood odor component. *Physiol. Behav.* 184 205–210. 10.1016/j.physbeh.2017.12.006 29223710

[B66] Lamb-Echegaray I. D. Noftz W. A. Stinson J. P. Gabriele M. L. (2019). Shaping of discrete auditory inputs to extramodular zones of the lateral cortex of the inferior colliculus. *Brain Struct. Funct.* 224 3353–3371. 10.1007/s00429-019-01979-6 31729553 PMC7088437

[B67] Lesicko A. M. H. Hristova T. S. Maigler K. C. Llano D. A. (2016). Connectional modularity of top-down and bottom-up multimodal inputs to the lateral cortex of the mouse inferior colliculus. *J. Neurosci.* 36 11037–11050. 10.1523/JNEUROSCI.4134-15.2016 27798184 PMC5098839

[B68] LeVay S. Wiesel T. N. Hubel D. H. (1980). The development of ocular dominance columns in normal and visually deprived monkeys. *J. Comp. Neurol.* 191 1–51. 10.1002/cne.901910102 6772696

[B69] Lewis F. C. Kelly L. (1974). Oral stereognosis and auditory discrimination by adults, summary. *Percept. Motor Skills* 38:1218. 10.2466/pms.1974.38.3c.1218 4417332

[B70] Liu M. Xie F. Dai J. Zhang J. Yuan K. Wang N. (2023). brain-wide inputs to the non-lemniscal inferior colliculus in mice. *Neurosci. Lett.* 793:136976. 10.1016/j.neulet.2022.136976 36427816

[B71] Loftus W. C. Malmierca M. S. Bishop D. C. Oliver D. L. (2008). The cytoarchitecture of the inferior colliculus revisited: A common organization of the lateral cortex in rat and cat. *Neuroscience* 154 196–205. 10.1016/j.neuroscience.2008.01.019 18313229 PMC2562950

[B72] Lorente de Nó R. (1938). “The cerebral cortex: architecture, intracortical connecrtions, motor projections,” in *Physiology of the Nervous System*, ed. Fulton J. F. (New York, NY: University of Oxford).

[B73] Malmierca M. S. Saint Marie R. L. Merchan M. A. Oliver D. L. (2005). Laminar inputs from dorsal cochlear nucleus and ventral cochlear nucleus to the central nucleus of the inferior colliculus: Two patterns of convergence. *Neuroscience* 136 883–894. 10.1016/j.neuroscience.2005.04.040 16344158

[B74] Manis P. B. (1989). Responses to parallel fiber stimulation in the guinea pig dorsal cochlear nucleus in vitro. *J. Neurophysiol.* 61 149–161. 10.1152/jn.1989.61.1.149 2918341

[B75] Masterton R. B. (1997). Neurobehavioral studies of the central auditory system. *Ann. Otol. Rhinol. Laryngol.* 106 31–34. 9153114

[B76] Masterton R. B. Granger E. M. (1988). Role of the acoustic striae in hearing: contributions of dorsal and intermediate striae to detection of noises and tones. *J. Neurophysiol.* 60 1841–1860. 10.1152/jn.1988.60.6.1841 2466961

[B77] Masterton R. B. Thompson G. C. Bechtold J. K. RoBards M. J. (1975). Neuroanatomical basis of binaural phase-difference analysis for sound localization: A comparative study. *J. Comp. Physiological Psychol.* 89 379–386. 10.1037/h0077034 1194445

[B78] McCrea R. A. Strassman A. May E. Highstein S. M. (1987). Anatomical and physiological characteristics of vestibular neurons mediating the horizontal vestibulo-ocular reflex of the squirrel monkey. *J. Comp. Neurol.* 264 547–570. 10.1002/cne.902640408 2824574

[B79] McGurk H. MacDonald J. (1976). Hearing lips and seeing voices. *Nature* 264 746–748. 10.1038/264746a0 1012311

[B80] McInturff S. Adenis V. Coen F. V. Lacour S. P. Lee D. J. Brown M. C. (2023). Sensitivity to Pulse Rate and Amplitude Modulation in an Animal Model of the Auditory Brainstem Implant (ABI). *J. Assoc. Res. Otolaryngol.* [Epub ahead of print]. 10.1007/s10162-023-00897-z 37156973 PMC10335994

[B81] McInturff S. Coen F. V. Hight A. E. Tarabichi O. Kanumuri V. V. Vachicouras N. (2022). Comparison of Responses to DCN vs. VCN Stimulation in a Mouse Model of the Auditory Brainstem Implant (ABI). *J. Assoc. Res. Otolaryngol.* 23 391–412. 10.1007/s10162-022-00840-8 35381872 PMC9085982

[B82] Merzenich M. M. Kitzes L. Aitkin L. (1973). Anatomical and physiological evidence for auditory specialization in the mountain beaver (aplodontia rufa). *Brain Res.* 58 331–344. 10.1016/0006-8993(73)90005-X 4756133

[B83] Merzenich M. M. Reid M. D. (1974). Representation of the cochlea within the inferior colliculus of the cat. *Brain Res.* 77 397–415. 10.1016/0006-8993(74)90630-1 4854119

[B84] Mesulam M. M. Hegarty E. Barbas H. Carson K. A. Gower E. C. Knapp A. G. (1980). Additional factors influencing sensitivity in the tetramethyl benzidine method for horseradish peroxidase neurohistochemistry. *J. Histochem. Cytochem.* 28 1255–1259. 10.1177/28.11.6159394 6159394

[B85] Middlebrooks J. C. (2015). Sound localization. *Handb. Clin. Neurol.* 129 99–116. 10.1016/b978-0-444-62630-1.00006-8 25726265

[B86] Milinkeviciute G. Muniak M. A. Ryugo D. K. (2017). Descending projections from the inferior colliculus to the dorsal cochlear nucleus are excitatory. *J. Comp. Neurol.* 525 773–793. 10.1002/cne.24095 27513294

[B87] Miller J. Basbaum A. I. (1975). Topography of the projection of the body surface of the cat to cuneate and gracile nuclei. *Exp. Neurol.* 49 281–290. 10.1016/0014-4886(75)90211-3 1183527

[B88] Moore J. K. Moore R. Y. (1971). A comparative study of the superior olivary complex in the primate brain. *Folia Primatol.* 16 35–51. 10.1159/000155390 4111530

[B89] Morest D. K. Oliver D. L. (1984). The neuronal architecture of the inferior colliculus in the cat: Defining the functional anatomy of the auditory midbrain. *J. Comp. Neurol.* 222 209–236. 10.1002/cne.902220206 6699208

[B90] Mugnaini E. Warr W. B. Osen K. K. (1980a). Distribution and light microscopic features of granule cells in the cochlear nuclei of cat, rat, and mouse. *J. Comp. Neurol.* 191 581–606. 10.1002/cne.901910406 6158528

[B91] Mugnaini E. Osen K. K. Dahl A. Friedrich V. L. Korte G. (1980b). Fine structure of granule cells and related interneurons (termed golgi cells) in the cochlear nuclear complex of cat, rat and mouse. *J. Neurocytol.* 9 537–570. 10.1007/bf01204841 7441303

[B92] Müller M. von Hünerbein K. Hoidis S. Smolders J. W. T. (2005). A physiological place-frequency map of the cochlea in the CBA/J mouse. *Hear. Res.* 202 63–73. 10.1016/j.heares.2004.08.011 15811700

[B93] Muniak M. A. Ayeni F. E. Ryugo D. K. (2018). Hidden hearing loss and endbulbs of Held: Evidence for central pathology before detection of ABR threshold increases. *Hear. Res.* 364 104–117. 10.1016/j.heares.2018.03.021 29598838

[B94] Muniak M. A. Mayko Z. M. Ryugo D. K. Portfors C. V. (2012). Preparation of an awake mouse for recording neural responses and injecting tracers. *J. Vis. Exp*. 64 3755. 10.3791/3755 22781848 PMC3476382

[B95] Muniak M. A. Rivas A. Montey K. L. May B. J. Francis H. W. Ryugo D. K. (2013). 3D model of frequency representation in the cochlear nucleus of the CBA/J Mouse. *J. Comp. Neurol.* 521 1510–1532. 10.1002/cne.23238 23047723 PMC3992438

[B96] Muniak M. A. Ryugo D. K. (2014). Tonotopic organization of vertical cells in the dorsal cochlear nucleus of the CBA/J Mouse. *J. Comp. Neurol.* 522 937–949. 10.1002/cne.23454 23982998 PMC3947158

[B97] Nelken I. Young E. D. (1994). Two separate inhibitory mechanisms shape the responses of dorsal cochlear nucleus type IV units to narrowband and wideband stimuli. *J. Neurophysiol.* 71 2446–2462. 10.1152/jn.1994.71.6.2446 7931527

[B98] Nelken I. Young E. D. (1996). Why do cats need a dorsal cochlear nucleus? *J. Basic Clin. Physiol. Pharmacol.* 7 199–220. 10.1515/jbcpp.1996.7.3.199 8910137

[B99] Newlands S. D. Perachio A. A. (2003). Central projections of the vestibular nerve: A review and single fiber study in the Mongolian gerbil. *Brain Res. Bull.* 60 475–495. 10.1016/s0361-9230(03)00051-0 12787868

[B100] Ohlemiller K. K. Jones S. M. Johnson K. R. (2016). Application of mouse models to research in hearing and balance. *J. Assoc. Res. Otolaryngol.* 17 493–523. 10.1007/s10162-016-0589-1 27752925 PMC5112220

[B101] Ohlrogge M. Doucet J. R. Ryugo D. K. (2001). Projections of the pontine nuclei to the cochlear nucleus in rats. *J. Comp. Neurol.* 436 290–303. 10.1002/cne.1068 11438931

[B102] Oliver D. L. (1984). Dorsal cochlear nucleus projections to the inferior colliculus in the cat: A light and electron microscopic study. *J. Comp. Neurol.* 224 155–172. 10.1002/cne.902240202 19180810

[B103] Oliver D. L. (1985). Quantitative analyses of axonal endings in the central nucleus of the inferior colliculus and distribution of ^3^H-labeling after injections in the dorsal cochlear nucleus. *J. Comp. Neurol.* 237 343–359. 10.1002/cne.902370306 4044892

[B104] Osen K. K. (1972). Projection of the cochlear nuclei on the inferior colliculus in the cat. *J. Comp. Neurol.* 144 355–371. 10.1002/cne.901440307 5027335

[B105] Park T. J. Grothe B. Pollak G. D. Schuller G. Koch U. (1996). Neural delays shape selectivity to interaural intensity differences in the lateral superior olive. *J. Neurosci.* 16 6554–6566. 10.1523/jneurosci.16-20-06554.1996 8815932 PMC6578907

[B106] Paxinos G. Franklin K. B. J. (2019). *The Mouse Brain in Stereotaxic Coordinates (Fifth Edition).* Oxford: Academic Press.

[B107] Pfeiffer R. R. (1966). Classification of response patterns of spike discharges for units in the cochlear nucleus: Tone-burst stimulation. *Exp. Brain Res.* 1 220–235. 10.1007/bf00234343 5920550

[B108] Portfors C. V. Mayko Z. M. Jonson K. Cha G. F. Roberts P. D. (2011). Spatial organization of receptive fields in the auditory midbrain of awake mouse. *Neuroscience* 193 429–439. 10.1016/j.neuroscience.2011.07.025 21807069

[B109] Portfors C. V. Roberts P. D. (2014). Mismatch of structural and functional tonotopy for natural sounds in the auditory midbrain. *Neuroscience* 258 192–203. 10.1016/j.neuroscience.2013.11.012 24252321

[B110] Qvist H. Dietrichs E. (1985). The projection from the superior colliculus to the lateral reticular nucleus in the cat as studied with retrograde transport of WGA-HRP. *Anat. Embryol.* 173 269–274. 10.1007/bf00316308 3002208

[B111] Qvist H. Dietrichs E. (1986). Afferents to the lateral reticular nucleus from the oculomotor region. *Anat. Embryol.* 175 261–269. 10.1007/bf00389604 2435193

[B112] Ramon-Moliner E. (1962). An attempt at classifying nerve cells on the basis of their dendritic patterns. *J. Comp. Neurol.* 1190 211–227. 10.1002/cne.901190207 13990676

[B113] Ramoìn y Cajal S. (1909). *Histologie du Systeme Nerveux de L’Homme et des Vertebres, 1.* Paris: Maloine, 10.5962/bhl.title.48637

[B114] Reiss L. A. Young E. D. (2005). Spectral edge sensitivity in neural circuits of the dorsal cochlear nucleus. *J. Neurosci.* 25 3680–3691. 10.1523/jneurosci.4963-04.2005 15814799 PMC6725373

[B115] Rhode W. S. Smith P. H. (1986). Physiological studies on neurons in the dorsal cochlear nucleus of cat. *J. Neurophysiol.* 56 287–307. 10.1152/jn.1986.56.2.287 3760922

[B116] Rhode W. S. Smith P. H. Oertel D. (1983). Physiological response properties of cells labeled intracellularly with horseradish peroxidase in cat dorsal cochlear nucleus. *J. Comp. Neurol.* 213 426–447. 10.1002/cne.902130407 6300199

[B117] Rice J. J. May B. J. Spirou G. A. Young E. D. (1992). Pinna-based spectral cues for sound localization in cat. *Hear. Res.* 58 132–152. 10.1016/0378-5955(92)90123-5 1568936

[B118] Robards M. J. (1979). Somatic neurons in the brainstem and neocortex projecting to the external nucleus of the inferior colliculus: An anatomical study in the opossum. *J. Comp. Neurol.* 184 547–565. 10.1002/cne.901840308 422756

[B119] Rockel A. J. Jones E. G. (1973). The neuronal organization of the inferior colliculus of the adult cat. I. The central nucleus. *J. Comp. Neurol.* 147 11–60. 10.1002/cne.901470103 4682181

[B120] Rose J. E. Greenwood D. D. Goldberg J. M. Hind J. E. (1963). Some discharge characteristics of single neurons in the inferior colliculus of the cat. I. Tonotopical organization, relation of spike-counts to tone intensity, and firing patterns of single elements. *J. Neurophysiol.* 26 294–320. 10.1152/jn.1963.26.2.294 13954634

[B121] Rossing T. D. (1982). *The science of sound*. Redding, MA: Addison-Welsey Publishing Co., Inc.

[B122] Roth G. L. Aitkin L. M. Andersen R. A. Merzenich M. M. (1978). Some features of the spatial organization of the central nucleus of the inferior colliculus of the cat. *J. Comp. Neurol.* 182 661–680. 10.1002/cne.901820407 721973

[B123] Rubio M. E. Gudsnuk K. A. Smith Y. Ryugo D. K. (2008). Revealing the molecular layer of the primate dorsal cochlear nucleus. *Neuroscience* 154 99–113. 10.1016/j.neuroscience.2007.12.016 18222048 PMC2493417

[B124] Ryugo D. K. Fekete D. M. (1982). Morphology of primary axosomatic endings in the anteroventral cochlear nucleus of the cat: A study of the endbulbs of held. *J. Comp. Neurol.* 210 239–257. 10.1002/cne.902100304 7142440

[B125] Ryugo D. K. Haenggeli C. Doucet J. R. (2003). Multimodal inputs to the granule cell domain of the cochlear nucleus. *Exp. Brain Res.* 153 477–485. 10.1007/s00221-003-1605-3 13680048

[B126] Ryugo D. K. Willard F. H. (1985). The dorsal cochlear nucleus of the mouse: A light microscopic analysis of neurons that project to the inferior colliculus. *J. Comp. Neurol.* 242 381–396. 10.1002/cne.902420307 2418077

[B127] Ryugo D. K. Willard F. H. Fekete D. M. (1981). Differential afferent projections to the inferior colliculus from the cochlear nucleus in the albino mouse. *Brain Res.* 210 342–349. 10.1016/0006-8993(81)90907-0 6164444

[B128] Saldaña H. M. Rosenblum L. D. (1993). Visual influences on auditory pluck and bow judgments. *Percept. Psychophys.* 54 406–416. 10.3758/bf03205276 8414899

[B129] Saleeba C. Dempsey B. Le S. Goodchild A. McMullan S. (2019). A Student’s guide to neural circuit tracing. *Front. Neurosci.* 13:897. 10.3389/fnins.2019.00897 31507369 PMC6718611

[B130] Saleeba C. Dempsey B. Le S. Goodchild A. McMullan S. (2020). Corrigendum: A Student’s guide to neural circuit tracing. *Front. Neurosci.* 14:177. 10.3389/fnins.2020.00177 32210751 PMC7076267

[B131] Scheibler E. Liu W. Weinandy R. Gattermann. (2005). Burrow systems of the mongolian gerbil (*Meriones unguiculatus Milne Edwards, 1867*). *Mammal. Biol.* 71 178–182. 10.1016/jmambio.2005.11.007

[B132] Schweitzer L. Cant N. B. (1985). Differentiation of the giant and fusiform cells in the dorsal cochlear nucleus of the hamster. *Dev. Brain Res.* 20 69–82. 10.1016/0165-3806(85)90088-4 4005620

[B133] Sekaran S. A. P. R. Lee C. P. Lim K. M. (2021). “Facial emotion recognition using transfer learning of Alexnet,” in *Proceedings of the 2021 9th International Conference on Information and Communication Technology (ICoICT)*, Melaka, 10.1109/icoict52021.2021.9527512

[B134] Sergeyenko Y. Lall K. Liberman M. C. Kujawa S. G. (2013). Age-related cochlear synaptopathy: an early-onset contributor to auditory functional decline. *J. Neurosci.* 33 13686–13694. 10.1523/JNEUROSCI.1783-13.2013 23966690 PMC3755715

[B135] Shokunbi M. T. Hrycyshyn A. W. Flumerfelt B. A. (1985). Spinal projections to the lateral reticular nucleus in the rat: A retrograde labelling study using horseradish peroxidase. *J. Comp. Neurol.* 239 216–226. 10.1002/cne.902390208 4044936

[B136] Shore S. E. Moore J. K. (1998). Sources of input to the cochlear granule cell region in the Guinea pig. *Hear. Res.* 116 33–42. 10.1016/s0378-5955(97)00207-4 9508026

[B137] Shore S. E. Zhou J. (2006). Somatosensory influence on the cochlear nucleus and beyond. *Hear. Res.* 21 90–99. 10.1016/j.heares.2006.01.006 16513306

[B138] Slee S. J. Young E. D. (2010). Sound localization cues in the marmoset monkey. *Hear. Res.* 260 96–108. 10.1016/j.heares.2009.12.001 19963054 PMC2819082

[B139] Sparks D. L. Hartwich-Young R. (1989). The deep layers of the superior colliculus. *Rev. Oculomot Res.* 3 213–255. 2486324

[B140] Spence C. Driver J. (2000). Attracting attention to the illusory location of a sound: reflexive crossmodal orienting and ventriloquism. *Neuroreport* 11 2057–2061. 10.1097/00001756-200006260-00049 10884070

[B141] Spirou G. A. May B. J. Wright D. D. Ryugo D. K. (1993). Frequency organization of the dorsal cochlear nucleus in cats. *J. Comp. Neurol.* 329 36–52. 10.1002/cne.903290104 8454725

[B142] Stamataki S. Francis H. W. Lehar M. May B. J. Ryugo D. K. (2006). Synaptic alterations at inner hair cells precede spiral ganglion cell loss in aging C57BL/6J MICE. *Hear. Res.* 221 104–118. 10.1016/j.heares.2006.07.014 17005343

[B143] Stebbings K. A. Lesicko A. M. H. Llano D. A. (2014). The auditory cortico collicular system: Molecular and circuit-level considerations. *Hearing Res.* 314, 51–59. 10.1016/j.heares.2014.05.004 PMC4140214 24911237

[B144] Stiebler I. Ehret G. (1985). Inferior colliculus of the house mouse. i. A quantitative study of tonotopic organization, frequency representation, and tone-threshold distribution. *J. Comp. Neurol.* 238 65–76. 10.1002/cne.902380106 4044904

[B145] Suthakar K. Ryugo D. K. (2017). Descending projections from the inferior colliculus to medial olivocochlear efferents: Mice with normal hearing, early onset hearing loss, and congenital deafness. *Hear. Res.* 343 34–49. 10.1016/j.heares.2016.06.014 27421755

[B146] Sutherland D. P. Masterton R. B. Glendenning K. K. (1998a). Role of acoustic striae in hearing: Reflexive responses to elevated sound-sources. *Behav. Brain Res.* 97 1–12. 10.1016/s0166-4328(98)00008-4 9867226

[B147] Sutherland D. P. Glendenning K. K. Masterton R. B. (1998b). Role of acoustic striae in hearing: Discrimination of sound-source elevation. *Hear. Res.* 120 86–108. 10.1016/s0378-5955(98)00056-2 9667434

[B148] Tamamaki N. Yanagawa Y. Tomioka R. Miyazaki J. Obata K. Kaneko T. (2003). Green fluorescent protein expression and colocalization with calretinin, parvalbumin, and somatostatin in the GAD67-GFP knock-in mouse. *J. Comp. Neurol.* 467 60–79. 10.1002/cne.10905 14574680

[B149] Virsu V. Oksanen-Hennah H. Vedenpää A. Jaatinen P. Lahti-Nuuttila P. (2008). Simultaneity learning in vision, audition, tactile sense and their cross-modal combinations. *Exp. Brain Res.* 186 525–537. 10.1007/s00221-007-1254-z 18183376

[B150] Walberg F. Dietrichs E. Nordby T. (1985). On the projections from the vestibular and perihypoglossal nuclei to the spinal trigeminal and lateral reticular nuclei in the cat. *Brain Res.* 333 123–130. 10.1016/0006-8993(85)90131-3 3995280

[B151] Weakley J. M. Kavusak E. K. Carroll J. B. Gabriele M. L. (2022). Segregation of multimodal inputs into discrete midbrain compartments during an early critical period. *Front. Neural Circuits* 16:882485. 10.3389/fncir.2022.882485 35463204 PMC9021614

[B152] Weinberg R. J. Rustioni A. (1987). A cuneocochlear pathway in the rat. *Neuroscience* 20 209–219. 10.1016/0306-4522(87)90013-3 3561761

[B153] Wightman F. L. Kistler D. J. (1989a). Headphone simulation of free-field listening. I: Stimulus synthesis. *J. Acoust. Soc. Am.* 85 858–867. 10.1121/1.397557 2926000

[B154] Wightman F. L. Kistler D. J. (1989b). Headphone simulation of free-field listening. II: Psychophysical validation. *J. Acoust. Soc. Am.* 85 868–878. 10.1121/1.397558 2926001

[B155] Williams I. R. Filimontseva A. Connelly C. J. Ryugo D. K. (2022). The lateral superior olive in the mouse: Two systems of projecting neurons. *Front. Neural Circuits* 16:1038500. 10.3389/fncir.2022.1038500 36338332 PMC9630946

[B156] Willott J. F. (1984). Changes in frequency representation in the auditory system of mice with age-related hearing impairment. *Brain Res.* 309 159–162. 10.1016/0006-8993(84)91022-9 6488006

[B157] Winer J. A. Miller L. M. Lee C. C. Schreiner C. E. (2005). Auditory thalamocortical transformation: structure and function. *Trends Neurosci*. 28 255–263. 10.1016/j.tins.2005.03.009 15866200

[B158] Wright D. D. Ryugo D. K. (1996). Mossy fiber projections from the cuneate nucleus to the cochlear nucleus in the rat. *J. Comp. Neurol.* 365 159–172. 10.1002/(SICI)1096-9861(19960129)365:1<159::AID-CNE12>3.0.CO;2-L 8821448

[B159] Wu C. Shore S. E. (2018). Multisensory activation of ventral cochlear nucleus d-stellate cells modulates dorsal cochlear nucleus principal cell spatial coding. *J. Physiol.* 596 4537–4548. 10.1113/jp276280 30074618 PMC6138285

[B160] Yang M. Augustsson H. Markham C. Hubbard D. Webster D. Wall P. (2004). The rat exposure test: A model of mouse defensive behaviors. *Physiol. Behav.* 81 465–473. 10.1016/j.physbeh.2004.02.010 15135018

[B161] Yin T. C. Chan J. C. (1990). Interaural time sensitivity in medial superior olive of cat. *J. Neurophysiol.* 64 465–488. 10.1152/jn.1990.64.2.465 2213127

[B162] Yost W. A. Pastore M. T. (2019). Individual listener differences in azimuthal front-back reversals. *J. Acoust. Soc. Am.* 146 2709–2715. 10.1121/1.5129555 31671982 PMC6814437

[B163] Yost W. A. Pastore M. T. Dorman M. F. (2020). Sound source localization is a multisystem process. *Acoust. Sci. Technol.* 41 113–120. 10.1250/ast.41.113 34305431 PMC8297655

[B164] Young E. D. Spirou G. A. Rice J. J. Voigt H. F. (1992). Neural organization and sreponses to complex stimuli in the dorsal cochlear nucleus. *Philos. Trans. R. Soc. Lond. B* 336 407–413. 10.1098/rstb.1992.0076 1354382

[B165] Zhan X. Ryugo D. K. (2007). Projections of the lateral reticular nucleus to the cochlear nucleus in rats. *J. Comp. Neurol.* 504 583–598. 10.1002/cne.21463 17701985

[B166] Zhou J. Shore S. E. (2006). Convergence of spinal trigeminal and cochlear nucleus projections in the inferior colliculus of the guinea pig. *J. Comp. Neurol.* 495 100–112. 10.1002/cne.20863 16432905

