# Supplementary figures and images for: Differential projections from the cochlear nucleus to the inferior colliculus in the mouse
Source: Front Neural Circuits. 2023 Jul 24;17:1229746. doi: 10.3389/fncir.2023.1229746 (PMC10405501; doi:10.3389/fncir.2023.1229746)

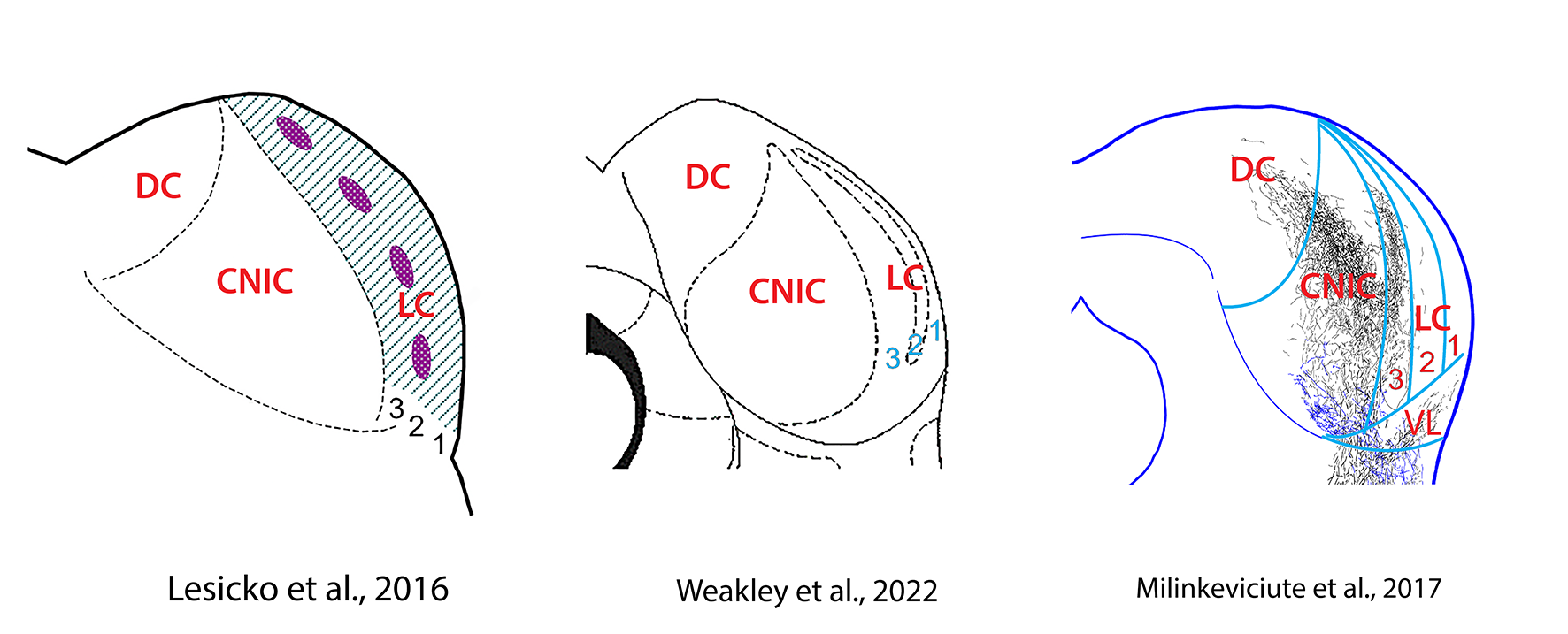

Supplement: Supplementary Figure 1 — Diagram of the main subdivisions of the mouse IC. The divisions follow the criteria established in an array of publications (Ryugo et al., 1981; Faye-Lund and Osen, 1985; Stebbings et al., 2014; Lesicko et al., 2016; Milinkeviciute et al., 2017; Weakley et al., 2022). The somatosensory modules (purple ovals, left image) are shown in layer 2 of the LC (middle image). The right image shows the traced fibers from the DCN (black) and VCN (blue) with the subdivisions. [file Image_1.TIF]

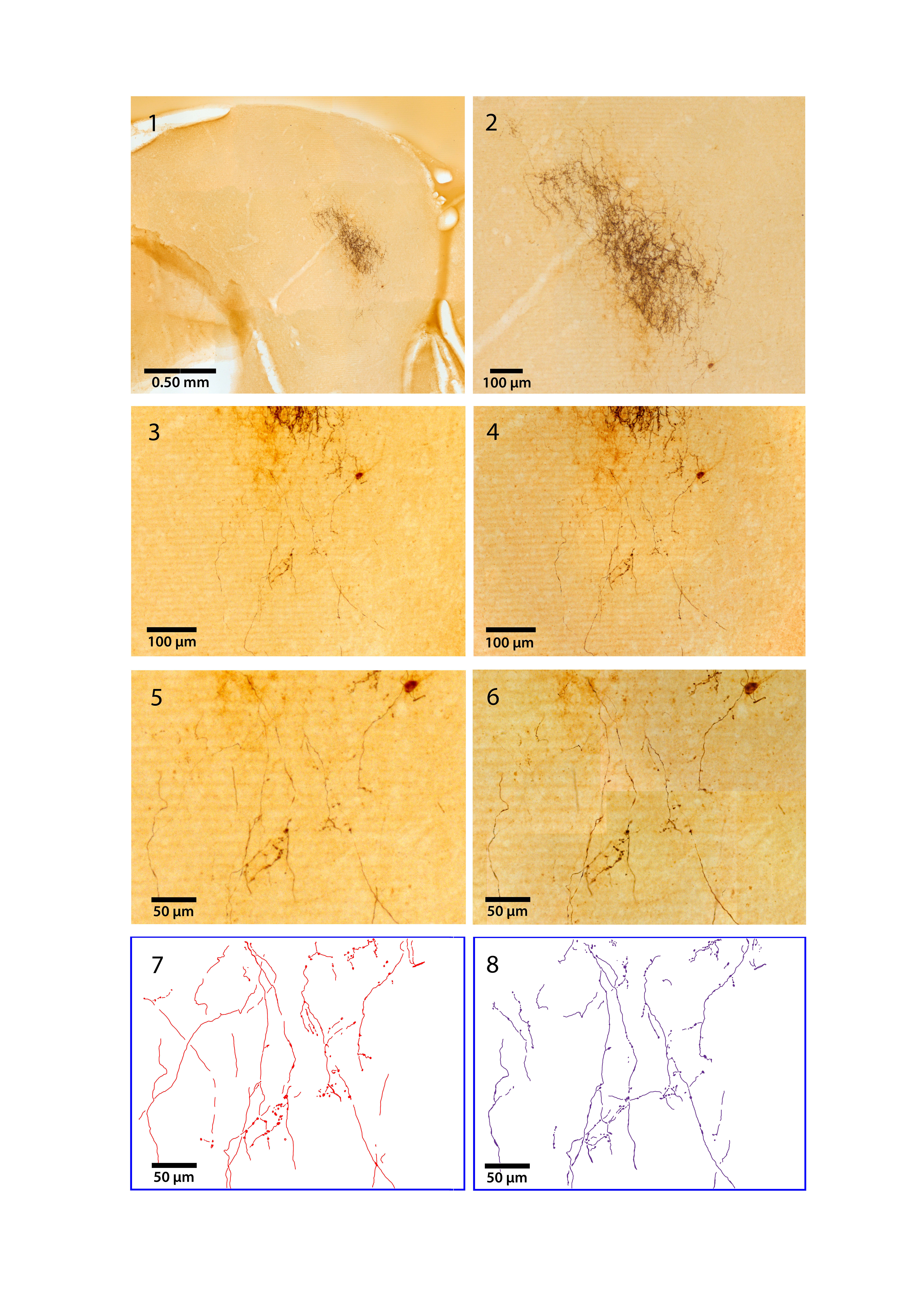

Supplement: Supplementary Figure 2 — This series of images review the process of fiber tracing for improving visualization of terminal patterns at low magnification. (Panel 1) Photographic montage of an IC section collected at 800 dpi using a 10× objective compared to a montage (Panel 2) of the same section collected using a 40× objective. (Panels 3–4) Each image was then digitally magnified so that labeled fibers and terminals in the IC could be manually drawn using a drawing tablet. We compared image collected with the 10× objective (Panel 5) to image collected using the 40× objective (Panel 6) and determined that qualitatively, there was not a major difference in terminal field plots when tracing the fibers photographed using a 10× or 40× objective (compare Panel 7 to Panel 8). These comparisons confirmed the utility of digitized magnification, the adequacy of 10× photomontages for terminal field plotting, and significantly reduced the amount of time needed to collect images for analysis. [file Image_2.JPEG]

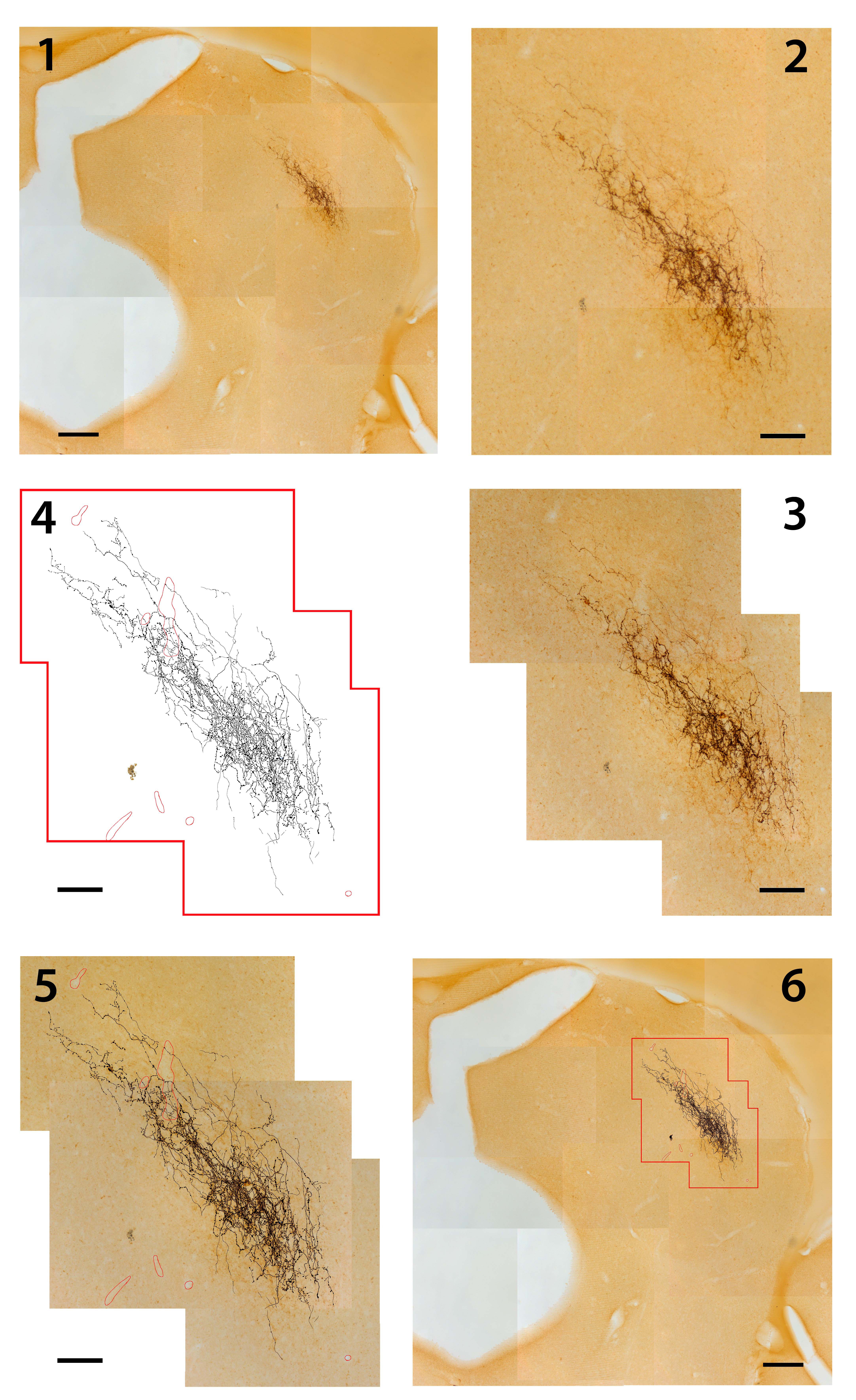

Supplement: Supplementary Figure 3 — This series of images illustrate how terminal fields were enhanced for low magnification viewing. Photographic tile sets that encompassed the entire IC across serial sections were collected using 10× objectives and were manually montaged (Panel 1) and then digitally magnified (Panel 2). The same field was photographed using a 20× objective (Panel 3). The digitized image was resampled at 800 dpi and then magnified again so that individually labeled fibers and terminals in the IC could be manually drawn (Panel 4). The drawings and the photomicrograph were merged (Panel 5), scaled, and repositioned back onto the photomontage made with the 10× objective (Panel 6). This procedure brought out details that were not apparent prior to the tracing (compare Panel 1 to Panel 6). This method enhanced the contrast and details of terminal field patterns. Scale bars: 250 μm for panels 1 and 6; 100 μm for panels 2–5. [file Image_3.JPEG]

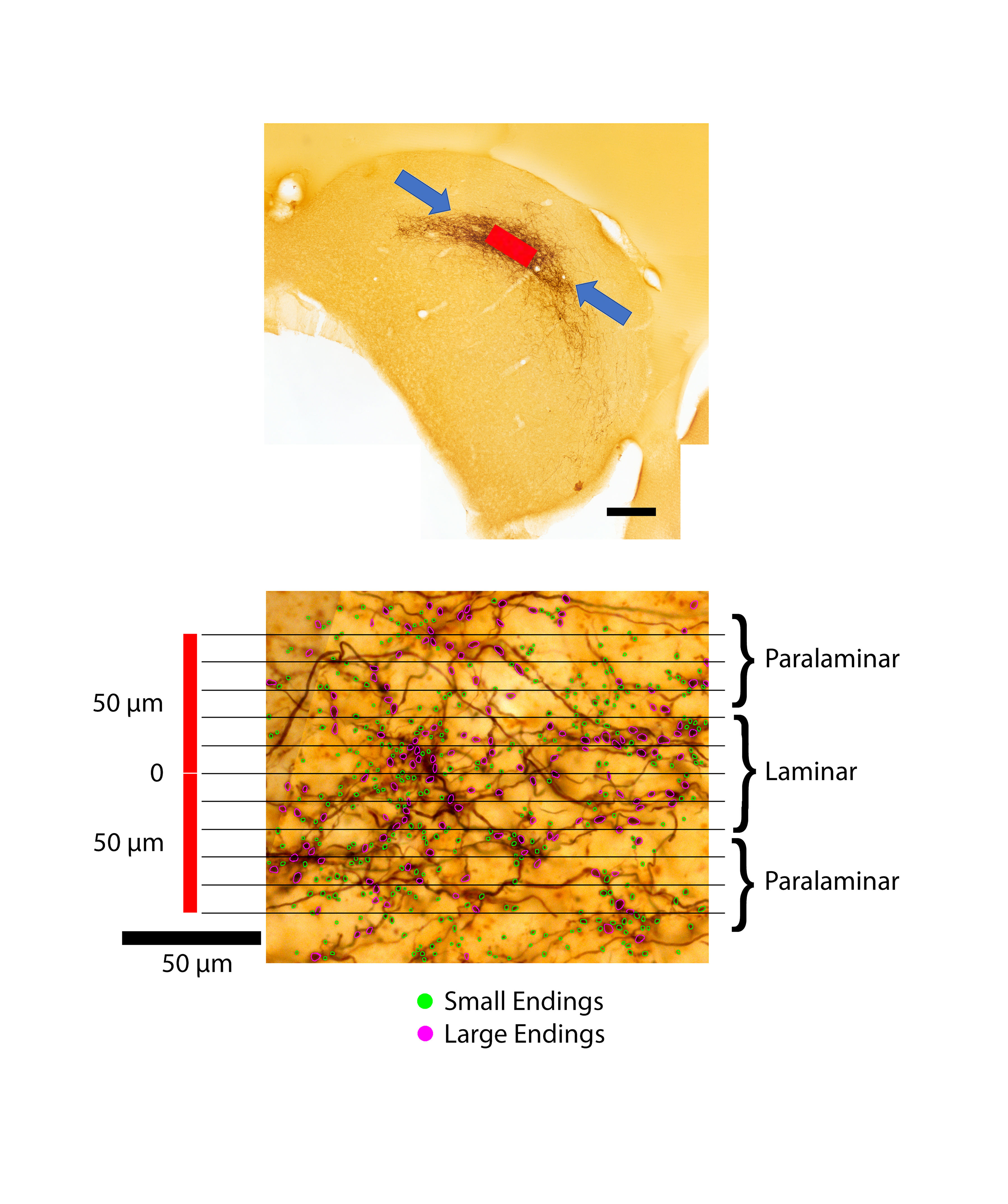

Supplement: Supplementary Figure 5 — This figure shows how we analyzed the distribution of terminal endings with respect to the pattern of laminar projections. (A) The DCN projections to the IC are demonstrated by the darkly stained fibers and terminals revealed by a chromogenic reaction using diaminobenzidine. The blue arrows flank the curved lamina and the red rectangle shows the sampling area. (B) The red box in panel (A) is shown where 0 approximates the middle of the lamina and represents the laminar “axis.” The horizontal lines are 10 μm apart. A visual assessment of terminal distribution is confirmed by counts, where the number of large and small terminals covaries together in each sector. The laminar axis is not highlighted by a preferential concentration of large terminals. Terminal ending density does diminish at the edges of the terminal field. [file Image_5.TIF]
