# Supplementary material for: Differential projections from the cochlear nucleus to the inferior colliculus in the mouse
Source: Front Neural Circuits. 2023 Jul 24;17:1229746. doi: 10.3389/fncir.2023.1229746 (PMC10405501; doi:10.3389/fncir.2023.1229746)
Supplement: Supplementary Table 1 — Summary table of all DCN and AVCN injections used in the study. [file Table_1.DOCX]

| **Animal ID** | **Strain** | **Injection Site** | **Tracer** | **BF (kHz)** |
| --- | --- | --- | --- | --- |
| AM217 | CBA/CaH | DCN | 10% BDA | 11 |
| AM219 | CBA/CaH | DCN | 10% BDA | 10 |
| AM246 | CBA/CaH | DCN | 10% BDA | 12 |
| AM289 | CBA/CaH | DCN | 10% DA Alexa555 | 12 |
| AM298 | CBA/CaH | DCN | 10% BDA | 40 |
| AM303 | CBA/CaH | DCN | 10% BDA | 20 |
| AM326 | CBA/CaH | DCN | 10% BDA | 8 |
| AM341 | CBA/CaH | DCN | 10% BDA | 27 |
| AM342 | CBA/CaH | DCN | 10% BDA | 16 |
| AM361 | CBA/CaH | DCN | 10% BDA | 20 |
| AM520 | CBA/CaH | DCN | 10% DA Alexa488 | 24 |
| AM527 | CBA/CaH | DCN | 10% DA Alexa488 | 35 |
| AM640 | CBA/CaH | DCN | 10% DA Alexa488 | 25 |
| AM1177 | CBA/CaH | DCN | 10% DA Alexa488 | 19 |
| AM1177 | CBA/CaH | DCN | 10% DA Alexa555 | 31 |
| AM225 | CBA/CaH | AVCN | 10% BDA | 20 |
| AM227 | CBA/CaH | AVCN | 10% BDA | 51 |
| AM231 | CBA/CaH | AVCN | 10% BDA | 55 |
| AM238 | CBA/CaH | AVCN | 10% BDA | 14 |
| AM240 | CBA/CaH | AVCN | 10% BDA | 19 |
| AM1237 | CBA/CaH | AVCN | 10% DA Alexa555 | 23 |
